# Early Biological Modulations Resulting from 1-Week Venlafaxine Exposure of Marine Mussels *Mytilus galloprovincialis* Determined by a Metabolomic Approach

**DOI:** 10.3390/metabo12030197

**Published:** 2022-02-22

**Authors:** Gaëlle Ramirez, Elena Gomez, Thibaut Dumas, David Rosain, Olivier Mathieu, Hélène Fenet, Frédérique Courant

**Affiliations:** 1 HydroSciences Montpellier, University of Montpellier, CNRS, IRD, Montpellier, France; gaelle.ramirez@umontpellier.fr (G.R.); maria-elena.gomez-hernandez@umontpellier.fr (E.G.); thibaut.dumas@umontpellier.fr (T.D.); david.rosain@umontpellier.fr (D.R.); o-mathieu@chu-montpellier.fr (O.M.); helene.fenet@umontpellier.fr (H.F.); 2 Laboratoire de Pharmacologie-Toxicologie, CHU de Montpellier, Montpellier, France

**Keywords:** antidepressant, aquatic organisms, effects, liquid chromatography-mass spectrometry, pharmaceuticals

## Abstract

There is growing evidence of the presence of pharmaceuticals in natural waters and their accumulation in aquatic organisms. While their mode of action on non-target organisms is still not clearly understood, their effects warrant assessment. The present study assessed the metabolome of the Mediterranean mussel (*Mytilus galloprovincialis*) exposed to a 10 µg/L nominal concentration of the antidepressant venlafaxine (VLF) at 3 time-points (1, 3, and 7 days). Over the exposure period, we observed up- or down-modulations of 113 metabolites, belonging to several metabolisms, e.g., amino acids (phenylalanine, tyrosine, tryptophan, etc.), purine and pyrimidine metabolisms (adenosine, cyclic AMP, thymidine, etc.), and several other metabolites involved in diverse functions. Serotonin showed the same time-course modulation pattern in both male and female mussels, which was consistent with its mode of action in humans, i.e., after a slight decrease on the first day of exposure, its levels increased at day 7 in exposed mussels. We found that the modulation pattern of impacted metabolites was not constant over time and it was gender-specific, as male and female mussels responded differently to VLF exposure.

## 1. Introduction

Venlafaxine (VLF) is an antidepressant of the serotonin (5-HT) and norepinephrine (NE) reuptake inhibitor family (SNRI). It is commonly detected in the aquatic environment [1,2] and particularly in marine waters, as demonstrated by its quantification ranging from some ng/L to a little hundred ng/L in coastal waters around the world [3,4,5,6,7]. Several studies have demonstrated that VLF bioaccumulates in marine organisms with levels from ng/g dry weight (dw) (*Mytilus galloprovincialis*, Mediterranean sea, France) to tens ng/g dw (*M. galloprovincialis*, Po delta, Italy) [8,9]. Some of its adverse effects have already been studied and highlighted in non-target aquatic organisms. The following disruptions caused by VLF exposure have been reported in fish species: impairment of embryo production and larval development, alterations in behavior, circadian rhythms, and liver metabolism [10,11,12]. At the molecular level, antidepressants modulate neurotransmitters in aquatic organisms [13]. According to its mode of action (MoA) in humans [14], VLF is thought to modulate 5-HT and NE; and, it could also affect them through other mechanisms [15]. At the molecular level, VLF effects are poorly documented; yet, this knowledge could help to assess its early effects and to explore its MoA in non-target organisms.

Environmental metabolomics is a recent science that is used to study organism–environment interactions and to assess organism disruptions at the molecular level. Metabolomics helps to reveal the impacts of a pollutant on an organism’s metabolism at a molecular level. It also highlights metabolite modulations triggered by a pollutant, which can help to identify its MoA in the studied organism [16]. Molecular studies also enable assessment of the effects of a xenobiotic to short-term exposure when effects at the individual level are not yet perceptible. The possibility of studying changes in a biological system over a time course at the molecular level opens many avenues toward gaining insight into how organisms deal with toxic substances [17]. Mass spectrometry is often used in metabolomics for semi-quantitative comparison of signal intensities detected in several groups of organisms, which can thereby highlight differential signals corresponding to endogenous metabolites. Metabolomics has already demonstrated its potential for studying MoA of xenobiotics and highlighting specific biomarkers of exposure [18]. It could therefore be interesting to study the response over time to differentiate between an adaptive response and a lasting modulation that may lead to an adverse effect of the xenobiotic on the organism. However, many studies have focused on a single-time when applying metabolomics [19,20,21]. A knowledge gap still exists concerning the response of an organism’s defence mechanisms to xenobiotic exposure, especially with regard to the early effects over time. 

Given that a large part of the world population lives close to sea coasts, and in many cases waste products are discharged into the environment through wastewater treatment plants, sea costs and marine water are under constant pressure from pollutants. Mussels—as organisms living in multiple environments according to their developmental stage [22]—are gaining increasing attention with regard to the issue of environmental contamination by pharmaceuticals. In addition, mussels filter large volumes of water and they are known as “suspension feeders”. They filter suspended materials and colloids and they are then known to efficiently bioaccumulate toxic compounds [23,24]. This species is therefore often used as a sentinel organism for the monitoring of coastal water pollution (mussel watch programs) [25]. Additionally, *Mytilus* species have been extensively used as model organisms to study the effects of pollutants in laboratory studies [26,27]. 

While very little information is available on the molecular effects of VLF in non-target organisms, the main objective of this study was to investigate the metabolites disrupted by VLF exposure in order to gain knowledge of the effects of such exposure in Mediterranean mussels (*Mytilus galloprovincialis*). Mussels were thus exposed for 1, 3, and 7 days to 10 µg/L of VLF. The metabolic fingerprints of control and exposed organisms were generated from the digestive glands of male and female mussels by liquid chromatography coupled with high resolution mass spectrometry (LC-HRMS). We evaluated temporal changes in disrupted metabolites along with their metabolic pathways. The sex-dependent response triggered by VLF exposure was also investigated. Finally, the VLF mode of action on *M. galloprovincialis* was investigated through the study of neurohormone alterations.

## 2. Results

### 2.1. Venlafaxine Quantification

Concentrations of VLF were measured throughout the experiment to control mussel exposure. The presence of VLF was not detected in control samples of both matrices (seawater and mussel tissues) and the mean concentration in spiked seawater was 11.0 ± 1.5 µg/L. In mussel soft tissues, mean VLF concentrations were 1.9 mg/kg dw and 2.5 mg/kg dw at days 3 and 7, respectively. A detailed description of these results and the detected VLF metabolites can be found in the study published by Ariza-Castro et al. (2021) [28].

### 2.2. Metabolite Modulation and Pathway Analysis

Analysis of the control and the exposed mussel digestive glands in ESI+ and ESI− led to an average of 6000 signals in male and female mussel samples. More than 70% of them showed an RSD lower than 30%. According to Want et al. (2013), these findings are sufficient to validate the variability in the present study [29]. For further processing, all signals with an RSD < 20% were considered, which allowed us to consider modulations of >20% relative to the control as relevant.

Among the 201 signals that could be annotated (a complete list of annotated signals is presented in Table S1), 113 showed a trend or significant up- or down-modulation over the course of exposure. These metabolites are presented in Table 1. For example, aspartylphenylalanine belongs to the alanine, aspartate, and glutamate metabolism (also to the phenylalanine metabolism). It was significantly up-modulated in males (+70%, *p* < 0.05) and down-modulated in female mussels (−38%, *p* < 0.05) on the first day of exposure.

**Figure 1:**
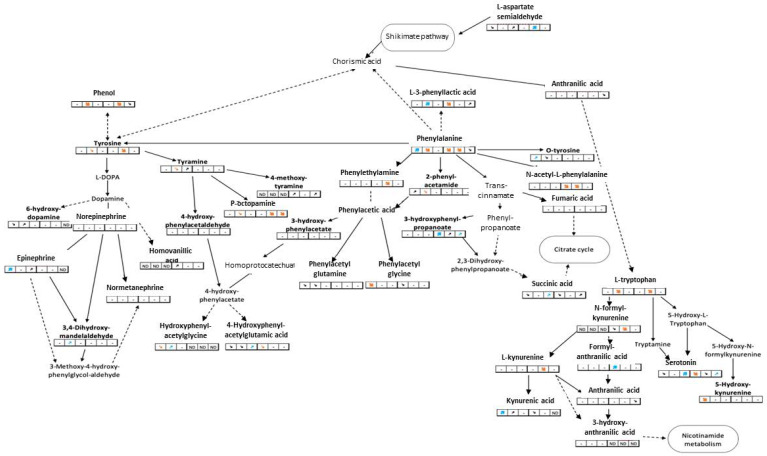
Partial metabolic pathways of tyrosine, phenylalanine, and tryptophan. Bold metabolites are those detected in our study, plain arrows between two metabolites represent direct links, dashed arrows represent incomplete links. Tables below metabolites are extracts of the Table 2: the first 3 boxes represent modulations in male mussels and the last 3 boxes concern female mussels. Colored arrows represent a trend or a significant modulation (when bold).

Twenty-one metabolic pathways had at least two significantly modulated metabolites over time, including phenylalanine, tyrosine, and tryptophan metabolic pathways. These latter three pathways (Figure 1) are known to be interrelated and to trigger the synthesis of many neurotransmitters such as catecholamines or 5-HT [30]. Nine tryptophan metabolites were annotated and eight of them were impacted by VLF, with 5-HT being one of them. This neurotransmitter showed the same modulation pattern in male and female mussels: down-modulated on the first day of exposure and then up-modulated on the seventh day.

### 2.3. Enrichment Analysis

Different metabolic pathways were found to be altered in male and female mussels as a function of time. The complete results of QEA are presented in Figures S1 and S2: a *p*-value was attributed for each pathway on the basis of the detailed influence of an individual compound to the total score of the metabolite set. Some of the pathways are inter-related such as the phenylalanine, tyrosine, and tryptophan metabolisms; or, they appear to be impacted several times for male or female mussels in which case a number in brackets was attributed to them in Table 2. For male mussels, the number of impacted pathways increased during VLF exposure: two single pathways tended to be disrupted on the first day, followed by 4, and then 9 significantly impacted pathways at days 3 and 7, respectively. Conversely, the number of impacted pathways seemed relatively homogenous for females (8, 12, and 11 pathways disrupted or showing a disruption trend on days 1, 3, and 7, respectively).

While some of the pathways showed latency and were only disrupted on the seventh day, e.g., the alanine, aspartate, and glutamate metabolisms, others appeared to be altered preferentially on the first days of exposure, such as the phenylalanine, tyrosine, and tryptophan metabolisms or the glycine, serine, and threonine metabolisms.

Other pathways appeared to be specific to male mussels such as vitamin B6 metabolism at day 3 or propanoate and histidine metabolisms at day 7, while others were only impacted in females, such as glycerophospholipid metabolism or pyrimidine metabolism at days 1 and 3 or purine metabolism at day 7.

## 3. Discussion

### 3.1. Early Responses after VLF Exposure

From the first day of exposure, several stress markers were noted in exposed mussels. A glutathione precursor, *L*-methionine was down-modulated in females’ digestive glands (−42%, *p* < 0.05 at day 1; −63%, *p* < 0.05 at day 3; and −40%, *p* > 0.1 at day 7). Glutathione is involved in detoxification processes via its oxidation to form oxidized glutathione to protect cells from oxidative damage [31]. Moreover, oxidized glutathione was up-modulated in female mussels on day 1, even though this result was not significant (+59%, *p* > 0.1) beside a significant up-modulation of cysteine-glutathione disulfide at days 1 and 3 (+94% and +70%, respectively, *p* < 0.05), supporting the hypothesis of elevated oxidative stress in female mussels [32]. Tetrahydrocortisol (steroid hormone biosynthesis) was up-modulated in male mussels on the first day of exposure (+49%, *p* < 0.05). This compound is a cortisol metabolite suspected to be released in response to stress, similar to the process in vertebrates [33,34,35]. Shi et al. (2019) also observed a cortisol increase in response to 10 days of exposure to 100 µg/L fluoxetine (FLX is an antidepressant) in blood clams (*T. granosa*) [36].

The branched-chain amino acid (BCAA) metabolism (valine, leucine, and isoleucine) was impacted by VLF exposure; e.g., *L*-isoleucine (or *L*-leucine) was down-modulated at days 1 and 7 in female mussels (−26%, *p* < 0.05 and −22%, *p* > 0.1, respectively). As they can be incorporated into proteins and used as substrates by the immune system, BCAAs are known to play a role in immunoregulation [37]. In bivalves, BCAAs have already been suggested to serve as biomarkers of immune stress after exposure to *V. harveyi* [38]. In humans, SNRIs and serotonin selective reuptake inhibitors (SSRI) were demonstrated to modify the activities of lymphocytes and monocytes and to have anti-inflammatory and immunosuppressive effects [39].

Exposure to VLF significantly modulated 5-HT. This metabolite is known to fulfill several functions in mollusks such as gill ciliary regulation [40,41], siphon opening, and muscle contraction [42]. By provoking muscle contraction, changes in 5-HT levels in a mussel’s digestive gland could lead to feeding behavior alterations. These disruptions may be manifested by decreased food intake, leading to decreased amino acid (AA) levels. Our study demonstrated that female mussels exposed to VLF had decreased AA levels, including *L*-lysine which was down-modulated at days 3 and 7 (−57%, *p* < 0.05 and −38%, *p* > 0.1, respectively), while *L*-threonine, *L*-tryptophan, and *L*-tyrosine were all down-modulated on the third day of exposure (−25%; −46% and −37%, respectively, *p* < 0.05). These results are consistent with an impact on feeding behavior very quickly after VLF exposure.

### 3.2. Sex-Dependent Responses

In humans, there is a growing number of studies highlighting sex-differences in responses between men and women to antidepressants. Differences in VLF metabolization were shown: women presented a lower O-desmethylvenlafaxine/VLF ratio than men for one of the studied enantiomers [43], although no significant sex differences were observable in response to VLF treatment for depression with melancholia [44]. On the contrary, during childhood, FLX did not lead to any pharmacokinetic differences between boys and girls [44]. However, in adulthood, women appeared to have better responses to FLX treatment than men (greater increases in tryptophan and decreases in cortisol concentrations) [45]. Women also had a higher risk of relapse after FLX treatment for major depression [46]. Sex-dependent differences in responses in humans suggested that venlafaxine could have a different impact on male and female mussels.

Indeed, the present study showed specific metabolites altered in male and female mussels in response to VLF exposure (28 were male-specific and 58 were female-specific). Moreover, the direction of metabolite variation (when common to both sexes) could differ, as for nicotinamide N-oxide (nicotinate and nicotinamide metabolism) that is significantly up-modulated in males and down-modulated in females on the seventh day of exposure. We also observed differences in the response times of metabolites; e.g., lactic acid showed an up-modulation trend on day 3 in females while this up-modulation did not appear before day 7 in male mussels. Among the altered pathways, the pyrimidine metabolism pathway differed between males and female. The QEA highlighted a specific impact in female mussels at days 1 and 3. For pyrimidine pathway metabolites, VLF exposure resulted principally in down-modulations in female mussels and up-modulations in males. Lipid metabolisms (glycerophospholipids, glycerolipids, and ether lipids) appeared to be more impacted in female mussels than in male (see QEA results).

Only a few metabolomic studies have explored sex-specific responses to a contaminant, although sex differences in response to contaminant exposure have been reported [38,47,48]. Such differences have already been highlighted in *M. galloprovincialis* when challenged by *V. harveyi* over a 24-h period [38]. Polar metabolites extracted from the digestive gland showed that male mussels responded mainly with signs of an enhanced energy demand (increased ATP levels and decreased glucose levels). Male mussels also presented higher AA levels (BCAA, threonine, alanine, arginine, and tyrosine), indicating changes in energy metabolism. In male mussels exposed to 10 µg/L 2,2′,4,4′-tetrabromodiphenyl ether (BDE 47) for 1 month, glutamate levels decreased and glucose along with 3 AA (threonine, lysine and histidine) increased, again suggesting energy metabolism disruptions [48]. Interestingly, we observed a synchronous increase of epinephrine, cAMP, tetrahydrocortisol, and AA levels at day 1 in male mussels (e.g., *L*-methionine and *L*-phenylalanine) without any common variation of this stress-induced catabolic pathway being observed in female mussels. Cortisol is a key enzyme for catabolism of protein to AA. Moreover, epinephrine (Epi) is the trigger neurohormone for stress response and cortisol and epinephrine work synergistically to increase energy pool. Finally, cyclic AMP (cAMP) is a second messenger for epinephrine. We can thus hypothesize the same kind of energetic metabolism disruptions for male mussels exposed to 10 µg/L of VLF.

Female mussels seem to respond to stressors by osmotic regulation disturbances, as reflected by osmolyte modulations [49]. When challenged by *V. harveyi*, taurine and betaine were depleted. When exposed to BDE 47, betaine and hypotaurine levels in mussels also changed. Glycine and alanine osmolytes were impacted in our study. Moreover, evidence of energy metabolism disruptions was observable in female mussels. Depletion of choline, phosphocholine, glycerophosphocholine, and ATP levels in vibrio-challenged female mussels are indicators of inhibitions in the conversions of choline and ATP into phosphocholine and ADP [38], which is consistent with energy metabolism disturbances. In our study, ATP was not detected but glycerophosphorylcholine (precursor of phosphocholine) showed a trend of up-modulation on days 1 and 3 (+29% and +25% respectively, *p* < 0.1) that could suggest a demand in phosphocholine following an inhibition in the conversion of choline and ATP. Moreover, AA levels in VLF-exposed female mussels mainly decreased, which could prompt a need for other metabolic sources to fulfill the increased energy demand to maintain homeostasis [50]. This theory was supported by the impacted lipid metabolisms in VLF-exposed females, as highlighted by the QEA.

Additionally, both male and female mussels presented energy metabolism disruptions, but those changes were the result of different mechanisms. Female mussels experienced osmoregulation disturbances that were not noted in male mussels. The early response to xenobiotic challenge may also be mainly chemical in female mussels with neutralization by glutathione, whereas a pharmacodynamic response involving the adrenergic pathway in link with the MoA of VLF seems to be involved in male mussels. As 5-HT modulation was observed in both sexes, males may be more sensitive to the adrenergic component of VLF.

### 3.3. Temporal Variation in the Effects

Gomez et al. (2021) studied the bioconcentration of VLF in marine mussels (*M. galloprovincialis*) and showed that mussels accumulate VLF following first order kinetics [51]. At 10 µg/L exposure concentration, VLF was quantified in mussel tissues at around 1200 ng/g dw on the first day of exposure, then increasing to more than 2000 ng/g dw on the seventh day of exposure. Clinical concentrations of venlafaxine are about hundreds ng/mL plasma; so, environmental exposure can lead mussels to reach pharmacological levels by extrapolation to humans. It could thus be hypothesized that different VLF effects could be observed over time before a steady state is reached. In addition, VLF metabolism (detoxification) was initiated over a 1-week period: different VLF-metabolite levels (dealkylation) appeared in mussel tissues over the 7-day exposure period.

In the present study, metabolite alterations were not constant over time. We did not see a clear and equivalent variation at the 3 time-points: among the 113 modulated metabolites, none varied in the same way over time, except for the poorly known metabolite vanillic acid 4-sulfate. A few studies have focused on time-dependent responses to pollutant exposure and they clearly highlighted modulation variations over time. When *M. galloprovincialis* was exposed to polystyrene microplastics (PM) for 24, 48, and 72 h, detected AA tended to increase during the first two days of exposure and then decreased on the third day (BCAA, alanine, dimethylglycine, and tyrosine), whereas glycine levels (a glutathione constituent) steadily decreased over time [52]. When exposed to 1 µg/L FLX for 10 days, eastern oysters (*C. virginica*) also showed the same pattern for 6 AA (alanine, aspartate, isoleucine, proline, threonine, and valine) [53]. In our study, this trend was noted for 2 AA: *L*-phenylalanine increased at day 1 in male mussels and then decreased (+31% at day 1 and −31% at day 3, *p* < 0.05), while females reacted with up-modulation of *L*-lysine level on day 1 followed by a decrease at days 3 and 7 (respectively +39%, *p* > 0.1; −57% and −38%, *p* < 0.1). These changes in AA levels suggest that protein catabolism enhancement is an early response to exposure, followed by protein synthesis activation in order to replace the damaged proteins when organisms become acclimated to pollutants [52].

Exposure to PM also induced energy metabolism disruptions, marked by alterations in glucose, glycogen, and lactate levels (following the AA trend) and malonate alterations [52]. *C. virginica* responded with alterations in succinate and citrate levels (decreased at day 1 and increased at day 5) and alterations in fatty acid content [53]. In our study, we also detected alterations in lactate and malonate (two intermediates of the Krebs cycle) even though those two compounds did not follow the same modulation trend, as previously reported. In female *M. galloprovincialis*, the QEA revealed that VLF exposure led to disturbances in glycerophospholipid, glycerolipid, and ether lipid metabolisms during the first three days of exposure. Taken together, these findings suggest a possible alteration in mussel energy metabolism as an early response, followed by a return to homeostasis. To our knowledge, our metabolomics study is the first attempt to explore time-dependent responses to pharmaceutical exposure in marine mollusks. Our results highlighted the interest of conducting this research to gain further insight into toxic events (or MoAs).

### 3.4. Mode of Action of VLF on M. galloprovincialis, Impact on Neurotransmitters

As an antidepressant of the SNRI class whose main mechanism is the inhibition of 5-HT and NE reuptake transporters on presynaptic neurons in the human brain [54], VLF was expected to impact neurotransmitter levels, particularly 5-HT and NE. The MoA of VLF is poorly documented on invertebrates. It is well established in humans that the initiation of a 5-HT reuptake-blocker treatment induces first, a decrease in 5-HT liberation (level) because of the reinforced stimulation of the presynaptic 5HT1A receptor that acts as modulator of 5-HT transmission and second, an increase in 5-HT transmission after prolonged treatment with a blocker for at least one month because of the desensitization of the presynaptic receptor. In contrast, the postsynaptic 5HT1A receptor undergoes no desensitization and this differential pattern is the background for the reinforcement of 5-HT neurotransmission on long term treatment with antidepressants [55,56]. While extrapolation from human pharmacology to mussels is tricky, there is a strong homology between the human 5HT1 receptor and the serotonin receptor of the mussel [57]. Regarding our results, VLF seems to act similarly in Mediterranean mussels. We observed that 5-HT decreased in male and female mussels on the first day of treatment (−36%, *p* > 0.1 and −63%, *p* < 0.05, respectively) and then increased during the seventh day (+334%, *p* < 0.05 and +95% *p* < 0.1, respectively). Although its down-modulation on the first day in male mussels was not significant, the modulation amplitude is high enough to corroborate that theory. As 5-HT is the only annotated compound that showed a similar modulation pattern in male and female mussels over time, the serotoninergic effect of VLF on mussels according to MoA is confirmed.

The presence of 5-HT has already been detected in bivalve gonads [58], and it could be responsible for oocyte maturation and germinal vesicle breakdown [59,60,61]; 5-HT and other 5-HT-receptor ligands are also known to induce spawning in marine bivalves [62]. Several studies have shown that exposure to antidepressants impairs reproductive processes in bivalves. Fong (1998) found that 50 nM of FLX and 1 nM of fluvoxamine (two SSRI) induced male spawning in zebra mussels (Dreissena polymorpha) [63]. Exposure to FLX at 20 ng/L also decreased numbers of oocites per follicule and spermatozoa per seminiferous tubules in gonads of zebra mussels [64], although this concentration was too low to induce spawning. Spermatogenesis involves 5-HT along with Epi; and, Epi is particularly suspected to have an impact on spermatozoa number and functionality [65]. Elevated levels of Epi in VLF-exposed male mussels at days 1 and 7 (+88%, *p* < 0.05 and +43%, *p* > 0.1, respectively) and changes in 5-HT levels could therefore have an impact on the reproductive function in *M. galloprovincialis*.

Otherwise, NE levels were not altered by VLF exposure in both sexes. Although Epi appeared to be up-modulated in male mussels, its levels remained unchanged in females. Then, VLF showed a serotoninergic effect but no clear adrenergic effect, particularly in female mussels, which was not expected. In humans, the VLF dose is known to influence the therapeutic effect. The in vitro affinity of venlafaxine has been reported to be higher for the 5-HT human transporter than for NE (Ki: 82 nM and 2480 nM, respectively) [66]. Thus, the main clinical effect of VLF at a low dose is that a serotoninergic and an adrenergic effect happens for higher doses [14]. This suggests that 10 µg/L exposure is in the low dose range for M. galloprovincialis, thereby explaining why only modulation in the serotoninergic system was observed. The presence and density of receptors weight the influence of the affinity on the pharmacodynamic response. A different pattern of distribution of 5-HT and NE receptors could explain the difference in effect between both sexes that have very differentiated physiological properties (5-HT and spawning and the importance of adrenergy mainly in the primordial phase of development). Another hypothesis is that the adrenergic transmission cascade in mussels is different from that in humans. Indeed, in mollusks, P-octopamine is considered to be a substitute for NE and it acts as a neurohormone, neuromodulator, and neurotransmitter [67]. In our study, this metabolite was significantly decreased in female mussels exposed to VLF on the third and the seventh days of the experiment (−37% and −33%, respectively, *p* < 0.05), and it was decreased in males on day 3 (−21%, *p* < 0.1). These results corroborate the theory that VLF adrenergic transmission cascade may differ from that observed in humans, and this trend warrants clarification in further studies.

The serotoninergic mode of action of VLF in humans has been confirmed in mussels. We may extrapolate that this mode of action could occur in other invertebrates.

## 4. Materials and Methods

### 4.1. Chemicals

Analytical pure standards were obtained from the three following suppliers: Sigma-Aldrich (now part of MERCK, Saint-Quentin-Fallavier, France), Santa Cruz Biotechnology (Santa Cruz, CA, USA), and Toronto Research Chemicals (Toronto, ON, Canada). Solvents (acetonitrile, formic acid, dichloromethane, and methanol) were obtained from CARLO ERBA Reagents (Val de Reuil, France) and were at least LC-MS-grade. Ultrapure water was generated by a Simplicity^®^ Water Purification System associated with a LC-Pak^®^ Polisher and a Millipak^®^ Express 20 filter (0.22 µm) from Merck Millipore (Bedford, MA, USA). 

### 4.2. Animals and Experimentation Design

*Mytilus galloprovincialis* mussels (*n* = 150) were obtained from a Mediterranean lagoon mussel farm (Bouzigues, France) in February 2017. The mussels were cleaned and uniformly selected according to their shell size (6.6 ± 0.29 cm, no significant difference between the different groups according to a Kruskal–Wallis test). They were randomly distributed into 30 glass aquaria (5 mussels per aquarium) with 2 L of filtered seawater (which was provided, filtered, sterilized, and degassed by IFREMER, Palavas, France). Following a 7-day acclimatization period, 15 aquaria underwent solvent control exposure (C, 20 µL methanol), and 15 aquaria underwent VLF exposure (E, 20 µL VLF solution at 1 µg/µL in methanol). We did not perform any negative controls since the final concentration of the solvent (0.01‰) was considered low enough to have no influence on the results [53,68,69]. Daily seawater renewal (static renewal) was carried out for each day of acclimatization and exposure periods. Aquaria pH (8.2 ± 0.1), temperature (14.6 ± 0.5 °C), salinity (34.7 ± 2.5 g/L), and oxygen concentration (9.9 ± 0.3 mg/L) were checked daily throughout the experiment. During the exposure period, VLF concentrations were reestablished every day after the seawater renewal and the feeding of mussels with the marine green alga *Tretraselmis suecica* (Greensea, Mèze, France) at a constant density (10,000 cells/mL). Daily seawater samples were taken to quantify VLF. Some of the mussels in the control group died during the exposure period (below 10% mortality) and were not replaced. On days 1, 3, and 7, mussels were collected for dissection and sex microscopy determination. The digestive glands and the remaining soft tissues were frozen at −80 °C prior to analysis. The digestive gland is one of the essential organs of mussels where xenobiotic metabolization occurs. Several studies conducted on the effect of xenobiotics on mussels successfully used digestive glands as the model tissue [70]. Some of the mussel digestive glands did not weigh enough to be analyzed according to our protocol.

Table 3 shows the number of mussels in each group. If present, numbers in brackets represent mussels that were dissected within the group and the other number represents mussels analyzed by the metabolomics approach. For example, there were 11 male mussels in the exposed group on day 1, but only 7 were analyzed because 4 had an insufficient sample size.

### 4.3. Metabolomics Sample Analysis

#### 4.3.1. Tissue Sample Preparation

After dissection, the digestive glands were freeze-dried and then ground into powder. To extract metabolites, 30 mg of each sample was homogenized in 240 µL of methanol (MeOH) and 75 µL of milliQ water was added and vortexed for 60 s. Then, 240 µL of dichloromethane (DCM) and 120 µL of milliQ water were added and further vortexed for 60 s. The samples were left on ice for 15 min then vortexed and centrifuged for 15 min at 2000× *g* at 4 °C. Then, 50 µL of the upper layer was transferred into glass tubes and dried under a nitrogen (N_2_) stream at 35 °C with a TurboVap^®^ LV concentration workstation from Caliper LifeSciences (Waltham, MA, USA). The extracts were re-suspended in 200 µL of a water/acetonitrile (ACN) mixture (95:5, *v*/*v*), transferred into centrifugal devices with a 10 kDa cut-off, and centrifuged at 10,000× *g* for 10 min. The filtrates were then transferred into 0.3 mL conic vials.

#### 4.3.2. Data Acquisition and Quality Control

Analyses were performed on a Thermo Scientific Vanquish^™^ Quaternary HPLC system (Waltham, MA, USA) coupled with a Thermo Scientific Q Exactive^™^ Focus Hybrid Quadrupole-Orbitrap mass spectrometer equipped with a heated electrospray ionization (HESI) probe. Metabolite separation was achieved using a reverse phase PFPP analytical column (100 mm × 2.1 mm; 3 µm particle size; Sigma Aldrich). Mobile phases (MP) were mixtures of water/ACN (99:1, *v*/*v*) (phase A) or water/ACN (1:99, *v*/*v*) (phase B) spiked with 0.1% of formic acid (FA). The flow rate was set at 250 µL/min, and a gradient elution was performed starting with 5% B and increasing from the 3rd min to 40% B during 5 min, then increasing to 50% B for 1 min, to finish at 95% B at the 18th min for 3 min. The gradient was allowed to return to starting conditions for 3 min followed by a 7-min re-equilibration period (total run time, 28 min).

The Q-Exactive^™^ Focus HRMS was turned to a mass resolving power of 35,000 with a mass range of 50–750 *m*/*z*. All samples were analyzed simultaneously in positive and negative electrospray ionization modes (ESI+ and ESI−). The analyses were completed with a spray voltage of 3.35 |kV|. The capillary and source temperatures were set at 300 °C.

A Quality Control (QC) sample corresponding to a pool of 88 µL of each sample extract was injected at the beginning of the injection sequence to equilibrate the column. To monitor the analytical repeatability and sensitivity during analysis, QC injections were repeated throughout the injection sequence. To assess the injection repeatability, relative standard deviations (RSD) were calculated for each signal detected in the QC samples. Only ions with an RSD lower than 20% were kept for data processing and annotation.

#### 4.3.3. Data Processing and Statistical Analysis

Due to the complexity of this dataset, data for female and male mussels were treated separately. The raw data were converted into mzXML files with MSConvert freeware (ProteoWizard 3.0 [71]). Then, the data were processed using the XCMS package in the R environment. Optimized XCMS parameters were implemented: *m*/*z* interval for peak picking was set at 0.002, the signal-to-noise ratio threshold was set at 10, the group bandwidth was set at 15, and the minimum fraction was set at 0.5. The data were first filtered, and signals presenting an RSD of QC > 20% and retention time beyond the analysis time range (60–1300 s) were discarded. After that, a Mann–Whitney test was performed daily to highlight signals presenting significant differences between the exposed and the control groups. Signals with a *p*-value < 0.1 and a difference amplitude >|20%| were selected for annotation and identification. The rationale for choosing such thresholds was based on (1) the low number of mussels per group, which may lead to a low statistical power, and (2) the opportunity to reveal a biological modulation trend due to exposure. Indeed, a trend (*p* < 0.1) toward up- or down-modulation of several metabolites from the same pathway may be more biologically relevant than a single marker metabolite (*p* < 0.05). Likewise, a modulation observed along the days of experimentation may also be more relevant than an isolated variation.

#### 4.3.4. Metabolite Annotation and Identification

Metabolite annotation was completed by mass-matching with 0.002 Da precision using the online Human Metabolome Database (HMDB, http://www.hmdb.ca/, 14 June 2021). The identification of annotated metabolites was based on two confirmation levels. The annotation levels are listed in Table 2 on the basis of the results reported by Sumner et al. 2007 [72]. Level 1 corresponds to identification confirmed by injection of the analytical standard (5 ng of standard) on the same analytical platform and under the same conditions (validation based on both accurate mass and retention times). Each standard was also added to QC samples to confirm the retention time within the matrix. In order to confirm the compound annotation, MS/MS acquisitions were also performed on QC samples with a high energy collision dissociation (HCD) cell set at 20 eV and a mass resolving power of 17,500. The MS/MS spectra observed after fragmentation of the signal of interest were compared to an in-house database. A metabolite’s identity was confirmed when most of the major fragments were in common. Level 2 metabolites were annotated on the basis of public databases (*m*/*z* matching and consistent physicochemical properties). The identified and annotated metabolites were assigned in metabolic pathways according to the Kyoto Encyclopedia of Genes and Genomes (KEGG, https://www.genome.jp/kegg/, 16 June 2021) and the pathway analysis online tool from MetaboAnalyst (https://www.metaboanalyst.ca/, 16 June 2021).

#### 4.3.5. Enrichment Analysis

After signal annotation, a quantitative enrichment analysis (QEA) was performed on Metaboanalyst 5.0 [73]. A table with all detected intensities of metabolites for each sampling day and for both sexes was loaded on Metaboanalyst. All compound labels were standardized with HMDB names and Pareto scaling was used. The KEGG pathway library was selected and we limited the results to the metabolite sets that contained at least two entries.

### 4.4. Venlafaxine Quantification

Tissue and water sample preparation conditions along with the VLF quantification method were described elsewhere [28].

#### 4.4.1. Sample Preparation

Briefly, 500 mg (±1 mg) dw of soft tissue samples obtained by pooling tissues of two individuals was weighed in a 50 mL polypropylene centrifuge tube and spiked with VLF-d6 (500 μg/kg). The tissues were then extracted by two sonication phases in 5 mL of extraction solvent (5% FA in MeOH) for 5 min followed by centrifugation at 23 °C (3400× *g*, 5 min). The resulting supernatants were combined; then, 0.5 g of lipid removal sorbent Z-Sep Plus and 5 mL of 5 mM ammonium acetate were added. The sample was then vortexed for 5 min and centrifuged at 23 °C (3400× *g*, 5 min). The extract obtained was transferred to a glass bottle (250 mL capacity) and diluted with milliQ water to a 150 mL final volume. After dilution, the sample pH was adjusted to 1.4 ± 0.1 then loaded at 1 mL/min onto the SPE Oasis MCX cartridges pre-conditioned with MeOH (6 mL), milliQ water (6 mL), and 2% FA in water (6 mL, pH: 1.40), respectively. The cartridges were allowed to dry, and then they were washed with 2% FA in water (6 mL, pH: 1.40), MeOH (2 mL), DCM (6 mL), and MeOH (3 mL). The analytes were eluted with 6 mL of 5% ammonium hydroxide in MeOH (5:95, *v*/*v*), and the solvent was evaporated to dryness under a gentle N_2_ stream at 35 °C. The residues were reconstituted with 200 μL of ACN/water (10/90, *v*/*v*). Finally, the sample was centrifuged at 13.4 × 103 rpm for 10 min to separate the residual lipids. The clear solution was transferred to a vial for LC-MS analysis.

A tailored version of the tissue extraction and cleaning method was applied to determine the real VLF concentration in seawater sampled during the mussel exposure period. Precisely, 18 mL of seawater was spiked with surrogate standard (VLF-d6 at 1 μg/L) and adjusted to pH 1.4 ± 0.1. A solid-phase extraction was conducted with the same method used for the tissues, except for the washing step, which was performed without DCM. The analyte was eluted with fresh prepared ammonium hydroxide in MeOH and the solvent was evaporated to dryness under an N_2_ stream. The residue was reconstituted in 200 μL of ACN/water (10/90, *v*/*v*) and transferred to a vial for LC-MS analysis.

#### 4.4.2. Data Acquisition

The analyses were run on the same equipment as for the metabolomics with a flow rate of 0.35 mL/min and a binary gradient of ACN (A) and water (B), both containing 0.1% FA as follows: 10% A at 0 min, 50% A at 4–6 min, 60% A at 8–10 min, 70% A at 14–16 min, 80% A at 17–19 min, 10% A at 21 min, and a stop time at 27 min. The data acquisition was performed simultaneously in ESI+ and ESI− with the parameters as follows: 275 °C capillary temperature; 200 °C heater temperature, and the electrospray voltage was set at 4.0/−4 kV. The full scan data were acquired at a mass resolution of 35,000 and an *m*/*z* scan range of 50 to 750. Using a mass inclusion list composed of the mass of the precursor ion of the compounds of interest, MS2 was achieved. A total of 10 eV absolute collision energy and 17,500 FWHM resolution were used.

#### 4.4.3. Identification and Quantification of VLF

Each compound was confirmed by comparison of its retention time and *m*/*z* with our in-house database. In order to quantify VLF in mussel soft tissues, calibration curves were plotted for blank mussel tissues by adding a fixed amount of the internal standards: VLF-d6 (500 μg/kg dw) and increasing quantities of VLF from 0 to 3000 μg/kg dw before extraction. In addition, a seawater calibration curve was plotted by adding a fixed amount of internal standard VLF-d6 (at 1 μg/L) and increasing quantities (0–15 μg/L) of the targeted VLF analyte in blank seawater.

## 5. Conclusions

The present study highlighted VLF-induced disruptions in several metabolites and metabolic pathways in *M. galloprovincialis*. Among them, the main impacted metabolites belonged to the metabolisms of several AA (phenylalanine, tryptophan, tyrosine, etc.), energy metabolism (e.g., malate), or they were markers of a stress response (e.g., oxidized glutathione, tetrahydrocortisol). Sex-dependent responses were also noted: male and female mussels showed several sex-specific impacted metabolites or differences in metabolite modulations. The findings suggest that male and female mussels have different protective mechanisms in response to stress, with one of these being the type of metabolites mobilized to fulfill an increased energy demand. The evaluation of VLF effects at 3 time-points allowed us to investigate the response patterns over time. Those results suggest that energy metabolism disruption is an early effect of VLF exposure. Among all the annotated metabolites, only 5-HT showed the same variation pattern in both male and female mussels, according to the MoA of VLF in humans. In mollusks, 5-HT is involved in several functions, such as reproduction and muscle constriction, which could be impacted by VLF exposure. An adrenergic effect was only observed in males in relation to a specific sensitivity also explainable by MoA, although a specific pharmacology in mussels may also exist. Further studies on pure adrenergic drug exposure in marine mussels are required to determine if adrenergic cascade differs between genders and between mussels and humans. The present study highlighted variations in the effects of VLF exposure over a 7-day period. It could be interesting to study the effects of a longer exposure so as to investigate the long-term effects of VLF on the metabolism of *M. galloprovincialis*. As already demonstrated in previous studies, the metabolomics approach was efficient in assessing the effects of VLF exposure on *M. galloprovincialis* at environmental concentrations.

## Figures and Tables

**Table 1:** Modulated metabolites sorted by their metabolic pathway (only pathways with at least 2 impacted metabolites are represented), for male and female mussels after 1, 3, and 7 days of exposure to VLF (†: metabolites present in several pathways; °: *L*-alanine and sarcosine could not be differentiated). Colored bold arrows represent significant modulations (*p* < 0.05; modulations > 20%), colored thin arrows represent a modulation trend (*p* < 0.1; modulations > 20%), and thin, black arrows are non-significant modulations (*p* > 0.1; modulations > 20%). Up arrows represent up-modulations and down arrows are for down-modulations. Dashes represent signals with a modulation < 20% and are considered as not modulated. ND: Not detected. RT: Retention time.

Pathway	Metabolite	Adduct	*m/z*	RT (min)	Male Mussels			Female Mussels			Annotation Level
					1	3	7	1	3	7	
Acetylcholine synthesis	Glycerophosphorylcholine	M+H+	258.1101	1.53	**-**	**-**	**-**	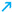	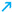	**-**	1
	Propionylcholine	M+	160.1337	4.46	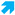	**↘**	**-**	**-**	**-**	**↗**	2
Alanine, aspartate and glutamate metabolism	Aspartylphenylalanine †	M+H+	281.1132	11.03	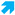	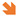	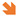	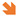	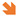	**↘**	2
	Gamma-l-glutamyl-l-leucine	M+H+	261.1445	9.63	**-**	**-**	**-**	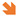	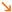	**↗**	1
	Isovalerylalanine	M+H+	174.1125	9.44	**-**	**-**	**-**	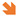	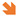	**-**	2
	*L*-Alanine †,°	M+H+	90.0546	1.45	**-**	**↘**	**-**	**-**	**-**	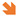	1
	*L*-Arginine †	M+H+	175.1190	2.63	**-**	**-**	**-**	**↗**	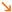	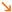	1
	N-acetyl-*L*-aspartic acid	M−H−	174.0408	1.48	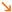	**-**	**↗**	**-**	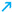	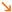	1
	N-Acetyl-*L*-glutamic acid	M−H−	188.0565	1.85	**-**	**-**	**-**	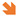	**-**	**-**	1
	Pyrrolidin-2-one †	M+Na+	108.0420	1.85	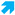	**-**	**↘**	**-**	**-**	**-**	1
	Succinic acid †	M−H−	117.0193	1.89	**↘**	**-**	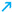	**↘**	**-**	**↗**	1
Amino Sugar Metabolism	Glucosamine	M+H+	180.0867	2.07	**-**	**-**	**-**	**-**	**-**	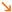	2
	N-Acetyl-*D*-glucosamine	M+FA−H−	266.0881	1.21	**-**	**-**	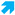	**-**	**-**	**↗**	1
	or N-Acetylmannosamine										
Arginine and Proline Metabolism and derivatives	2-Oxoarginine	M−H_2_O−H−	154.0617	1.92	**-**	**-**	**-**	**-**	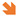	**-**	2
	4-(Glutamylamino) butanoate	M+H+	233.1132	3.13	**-**	**-**	**-**	**-**	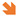	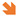	2
	Gamma-glutamyl-*L*-putrescine	M−H_2_O+H+	200.1388	3.94	**↗**	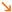	**-**	**-**	**↘**	ND	2
	*L*-4-Hydroxyglutamate semialdehyde	M−H_2_O−H−	128.0348	1.56	**-**	**↘**	**-**	**-**	**↗**	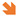	2
	*L*-Arginine †	M+H+	175.1190	2.63	**-**	**-**	**-**	**↗**	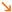	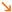	1
	*L*-Aspartate-semialdehyde †	M−H−	116.0353	1.44	**↘**	**-**	**↗**	**-**	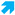	**-**	2
	*L*-Glutamine †	M+H+	147.0764	1.26	**-**	**↘**	**-**	**-**	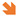	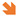	2
	N-alpha-acetylcitrulline	M−H−	216.0990	1.47	ND	ND	ND	ND	ND	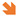	1
	Pyrrolidin-2-one †	M+Na+	108.0420	1.85	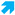	**-**	**↘**	**-**	**-**	**-**	1
	Sarcosine †,°	M+H+	90.05495	1.45	**-**	**↘**	**-**	**-**	**-**	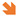	1
	Succinic acid †	M−H−	117.0193	1.89	**↘**	**-**	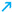	**↘**	**-**	**↗**	1
	Symmetric dimethylarginine	M+H+	203.1503	3.45	**-**	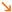	**↘**	**-**	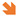	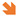	2
beta-Alanine metabolism	Hydroxypropionic acid †	M−H−	89.0244	1.55	**-**	**-**	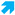	**-**	**-**	**↘**	2
	Pyrrolidin-2-one †	M+Na+	108.0420	1.85	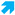	**-**	**↘**	**-**	**-**	**-**	1
	Uracil †	M−H−	111.0200	1.52	**-**	**-**	**-**	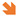	**-**	**-**	1
Biotin metabolism	*L*-Lysine †	M+H+	147.1128	2.50	**-**	**↘**	**-**	**-**	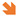	**↘**	1
	Pimelic acid	M−H−	159.0663	8.54	**↗**	**↘**	**-**	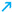	**↗**	**-**	1
Cysteine and methionine metabolism	2-Hydroxyphenethylamine	M−H_2_O+H+	120.0802	7.81	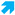	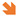	**-**	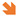	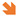	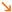	2
	*L*-Alanine †	M+H+	90.0550	1.45	**-**	**↘**	**-**	**-**	**-**	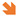	1
	*L*-Aspartate-semialdehyde †	M−H−	116.0353	1.44	**↘**	**-**	**↗**	**-**	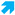	**-**	2
	*L*-Methionine †	M+H+	150.0583	2.57	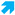	**-**	**-**	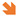	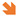	**↘**	1
Glutathione metabolism	5-l-glutamyl-l-alanine	M+H+	219.0976	1.69	**-**	**-**	**-**	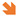	**-**	ND	2
	Cysteineglutathione disulfide	M−H−	425.0806	1.30	ND	ND	ND	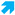	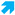	ND	2
	Pyroglutamic acid	M−H−	128.0353	1.56	**-**	**↘**	**-**	**-**	**↗**	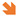	1
Glycine, serine and threonine metabolism	Aminoacetone	M+FA−H−	118.0510	1.33	**-**	**-**	**-**	**-**	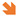	**-**	2
	*DL*-2-hydroxybutyric acid	M−H−	103.0401	2.09	**↗**	**-**	**-**	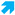	**-**	**-**	1
	Glycine †	M+H+	76.0393	1.34	**↘**	**-**	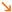	**-**	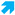	**-**	2
	Hydroxypyruvic acid	M−H−	103.0037	1.52	**-**	**-**	**-**	**↗**	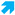	**↘**	2
	*L*-allothreonine	M−H−	118.0510	1.29	**-**	**-**	**-**	**-**	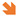	**-**	1
	*L*-Arginine †	M+H+	175.1190	2.63	**-**	**-**	**-**	**↗**	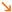	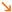	1
	*L*-Aspartate-semialdehyde †	M−H−	116.0353	1.44	**↘**	**-**	**↗**	**-**	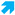	**-**	2
	*L*-Homoserine	M+H+	120.0655	1.38	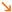	**↘**	**-**	**-**	**-**	**-**	1
	*L*-Methionine †	M+H+	150.0583	2.57	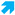	**-**	**-**	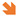	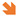	**↘**	1
	*L*-Tryptophan †	M+H+	205.0972	11.58	**-**	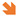	**-**	**-**	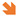	**-**	1
	N-Acetyl-*L*-threonine	M−H−	160.0615	1.50	**↘**	**-**	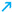	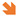	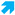	**-**	1
Histidine metabolism	Carnosine	M+H+	227.1139	3.56	ND	ND	ND	**-**	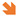	ND	2
	Histamine	M+H+	112.0869	5.84	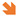	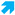	**↗**	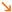	**↘**	**↗**	1
	Methylimidazoleacetic acid	M+H+	141.0659	3.10	**-**	**-**	**-**	**-**	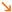	ND	1
Lysine biosynthesis or degradation	Aminoadipic acid	M+H+	162.0761	1.59	**-**	**-**	**-**	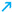	**-**	**-**	1
	Glutaric acid	M−H−	131.0350	2.48	**↗**	**-**	**-**	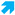	**↗**	**-**	1
	*L*-Aspartate-semialdehyde	M−H−	116.0353	1.44	**↘**	**-**	**↗**	**-**	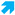	**-**	2
	*L*-Lysine †	M+H+	147.1128	2.50	**-**	**↘**	**-**	**-**	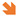	**↘**	1
	N-Succinyl-2-amino-6-ketopimelate	M+H+	290.0870	2.24	**-**	**↘**	**-**	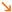	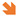	**-**	2
	N6-Acetyl-*L*-lysine	M+H+	189.1234	1.89	**-**	**-**	**↗**	**-**	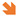	**-**	1
	Pipecolic acid	M+H+	130.0863	2.04	**↗**	**-**	**↗**	**↘**	**↘**	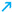	1
Nicotinate and Nicotinamide Metabolism	Niacinamide	M+H+	123.0553	2.46	**↗**	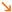	**-**	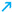	**-**	**-**	1
	Nicotinamide N-oxide	M+H+	139.0502	3.46	**↗**	**-**	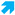	**-**	**-**	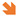	2
	Pyrrolidin-2-one †	M+Na+	108.0420	1.85	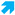	**-**	**↘**	**-**	**-**	**-**	1
	Trigonelline	M+H+	138.0550	1.45	**-**	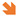	**-**	**-**	**-**	**-**	2
Pentose phosphate pathway	Deoxyribose	M+FA−H−	179.0561	1.21	**-**	**↗**	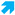	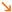	**-**	**-**	2
	Gluconic acid	M−H−	195.0510	1.15	**-**	**-**	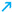	**-**	**-**	**↘**	1
	Ribose 1-phosphate †	M−H_2_O+H+	213.0153	2.93	**↗**	**-**	**-**	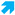	**↗**	**-**	2
Phenylalanine metabolism	2-Phenylacetamide	M+H+	136.0757	7.97	**↗**	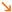	**-**	**-**	**-**	**-**	1
	3-(3-Hydroxyphenyl)propanoic acid	M−H−	165.0557	10.79	**-**	**-**	**-**	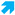	**↗**	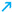	1
	Aspartylphenylalanine †	M+H+	281.1132	11.03	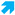	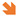	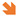	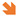	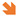	**↘**	2
	*L*-3-phenyllactic acid	M−H−	165.0557	10.48	**-**	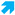	**-**	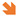	**-**	**↗**	1
	*L*-Phenylalanine	M+H+	166.0863	7.81	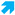	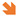	**-**	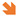	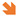	**↘**	1
	*L*-Tyrosine †	M+H+	182.0812	3.54	**-**	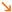	**-**	**-**	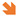	**-**	1
	N-Acetyl-*L*-phenylalanine	M−H−	206.0823	10.66	**-**	**-**	**-**	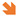	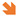	**-**	1
	Phenylacetylglycine	M−H−	192.0666	9.53	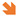	**-**	**-**	**↘**	**-**	**-**	1
	Phenylethylamine	M+H+	122.0964	13.93	**-**	**-**	**-**	**-**	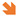	**-**	1
Purine metabolism	2-Hydroxyadenine	M+Na+	174.0386	2.34	**↗**	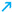	**-**	**-**	**-**	**↗**	2
	2′-Deoxyguanosine	M+H+	268.1040	2.27	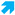	**↘**	**-**	**↘**	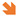	ND	1
	Cyclic AMP	M−H−	328.0452	1.51	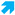	**-**	**-**	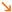	**↘**	**↘**	1
	Glycine †	M+H+	76.0393	1.34	**↘**	**-**	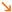	**-**	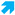	**-**	2
	Guanine	M+H+	152.0567	2.33	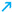	**-**	**-**	**-**	**-**	**-**	1
	Inosine-5′-monophosphate	M−H−	347.0398	1.18	**-**	**-**	**↗**	**-**	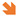	ND	1
	Ribose 1-phosphate †	M−H_2_O+H+	213.0153	2.93	**↗**	**-**	**-**	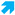	**↗**	**-**	2
	Succinyladenosine	M+H+	384.1150	3.60	**-**	**↗**	**-**	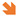	**-**	**↘**	2
	Xanthine	M−H−	151.0262	1.64	**-**	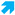	**-**	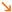	**-**	**-**	1
Pyrimidine metabolism	5-Methylcytosine	M−H−	124.0516	2.31	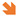	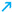	**↗**	**↗**	**-**	**-**	2
	Cytosine	M+H+	112.0505	2.41	**-**	**↗**	**-**	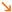	**-**	**-**	1
	Deoxycytidine	M+H+	228.0979	3.10	**-**	**-**	**↘**	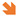	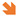	**-**	1
	Deoxyinosine	M−H−	251.0786	1.87	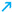	**↘**	**↘**	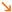	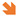	**↘**	1
	Dihydrothymine	M+H+	129.0659	1.97	**↘**	**↘**	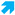	**-**	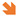	**-**	1
	Hydroxypropionic acid †	M−H−	89.0244	1.55	**-**	**-**	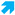	**-**	**-**	**↘**	1
	*L*-Glutamine †	M+H+	147.0764	1.26	**-**	**↘**	**-**	**-**	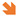	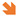	1
	Pseudouridine	M−H−	243.0623	1.31	ND	ND	ND	**-**	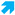	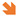	2
	Thymidine	M+FA−H−	287.0885	2.39	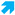	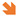	**-**	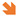	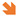	**-**	1
	Uracil †	M−H−	111.0200	1.52	**-**	**-**	**-**	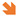	**-**	**-**	1
	*DL*-Lactic Acid †	M−H−	89.0244	6.38	**-**	**-**	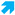	**-**	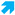	**↘**	1
Steroid hormone biosynthesis	4-Methylpentanal	M+ACN+H+	142.1226	10.07	**-**	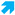	**-**	**-**	**-**	**-**	2
	Cortolone	2M+H+	227.1251	13.07	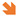	**-**	**-**	**-**	**-**	**-**	1
	Tetrahydrocortisol	M+FA−H−	411.2388	12.24	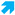	**-**	**-**	**-**	**-**	**-**	1
Tryptophan metabolism	5-Hydroxykynurenine	M+H+	225.0870	8.49	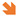	**-**	**-**	**-**	**-**	**-**	2
	*DL*-kynurenine	M+H+	209.0921	8.73	**-**	**-**	**-**	**-**	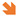	**-**	1
	Formylanthranilic acid	M−H−	164.0353	11.46	**-**	**-**	**-**	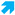	**-**	**-**	1
	Indoleacrylic acid	M+H+	188.0706	11.58	**-**	**-**	**-**	**-**	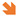	**-**	2
	Kynurenic acid	M+H+	190.0499	10.27	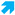	**↗**	**-**	**↘**	**-**	ND	1
	*L*-Tryptophan †	M+H+	205.0972	11.58	**-**	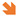	**-**	**-**	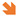	**-**	1
	N′-Formylkynurenine	M−H−	235.0724	7.23	ND	ND	ND	**↘**	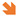	**-**	1
	Serotonin	M+H+	177.1022	10.46	**↘**	**-**	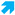	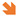	**↘**	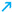	1
Tyrosine metabolism	1,2-Dehydrosalsolinol	M+H+	178.0863	9.84	**↗**	**↗**	**-**	**↗**	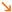	**↗**	2
	3,4-dihydroxymandelaldehyde	M−H−	167.0350	4.49	**-**	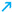	**-**	**-**	**-**	**-**	1
	4-Hydroxyphenylacetylglutamic acid	M+H+	282.0972	8.24	**↘**	**↘**	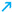	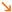	**-**	**-**	2
	Epinephrine	M+H+	184.0968	3.67	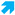	**-**	**↗**	**-**	**-**	ND	1
	Gamma-glutamyltyrosine	M−H−	309.1092	6.98	**-**	**-**	**-**	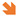	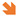	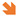	1
	Hydroxyphenylacetylglycine	M−H−	208.0615	5.02	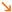	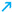	**-**	ND	ND	ND	1
	*L*-Tyrosine †	M+H+	182.0812	3.54	**-**	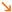	**-**	**-**	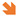	**-**	1
	N-Acetyl-*L*-tyrosine	M−H−	222.0772	7.29	**-**	**-**	**-**	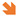	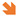	**-**	1
	O-tyrosine	M+H+	182.0812	7.40	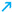	**↘**	**-**	**-**	**-**	**-**	1
	P-octopamine	M−H_2_O+H+	136.0751	3.54	**-**	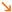	**-**	**-**	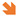	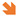	1
	Phenol	M−H_2_O+H+	77.0380	7.81	**-**	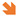	**-**	**-**	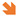	**↘**	2
	Tyramine	M+H+	138.0913	8.11	**-**	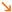	**↗**	**-**	**-**	**-**	1
Valine, leucine and isoleucine metabolism and derivatives	D*L*-isoleucine	M+H+	132.1019	3.92	**-**	**-**	**-**	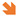	**-**	**↘**	2
	or *L*-Alloisoleucine										
	or *L*-Isoleucine										
	or *L*-Leucine										
	Erythronilic acid	M−H_2_O−H−	99.0446	2.71	**↗**	**↘**	**-**	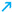	**-**	**-**	2
	Hydroxyisocaproic acid	M−H−	131.0714	8.74	**-**	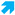	**-**	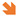	**↘**	**-**	1
	*L*-Threonine †	M−H−	118.0510	1.31	**-**	**-**	**-**	**-**	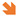	**-**	1
	N-acetylisoleucine	M−H−	172.0979	9.44	**-**	**-**	**-**	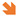	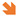	**-**	1
	N-Acetylvaline	M−H−	158.0823	4.80	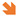	**-**	**-**	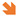	**-**	**-**	1
Vitamin B6 metabolism	4-Pyridoxic acid	M−H−	182.0460	1.44	**-**	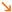	**-**	**-**	**-**	**-**	1
	5-Pyridoxolactone	M−H−	164.0353	3.25	**-**	**-**	**-**	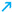	**-**	**-**	2
	*L*-Glutamine †	M+H+	147.0764	1.26	**-**	**↘**	**-**	**-**	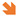	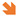	1
	Pyridoxine	M−H_2_O−H−	150.0555	10.81	**-**	**-**	**-**	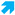	**-**	**↗**	2
Other metabolites	2-Ketohexanoic acid	M+H+	131.0703	3.56	**-**	**-**	**-**	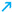	**-**	**-**	2
	*DL*-Lactic Acid †	M−H−	89.0244	6.38	**-**	**-**	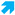	**-**	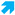	**↘**	1
	Ethyladipic acid	M−H−	173.0819	10.35	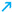	**↘**	**-**	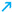	**↗**	**-**	2
	g-glutamyl-ornithine	M−H_2_O+H+	244.1286	3.19	ND	ND	ND	**-**	**-**	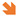	2
	*L*-Acetylcarnitine	M+H+	204.1230	5.78	**-**	**-**	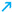	**↘**	**-**	**-**	1
	O-Propanoyl-*D*-carnitine	M+H+	218.1387	10.38	**↗**	**↗**	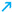	**↗**	**-**	**↘**	2
	Salsoline-1-carboxylate	M+H+	238.1074	9.94	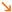	**-**	**-**	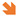	**-**	**-**	2
	Vanillic acid 4-sulfate	M+FA−H−	293.1143	10.65	**-**	**-**	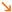	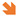	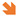	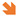	2

**Table 2:** Impacted metabolic pathways over time, sorted by decreasing *p*-value. When a group of related pathways was impacted more than one day or in one sex, it was attributed a number in brackets. Pathways in italics are specific to male mussels while bold pathways are specific to females. *: significantly impacted pathways; †: pathways that tended to be disrupted.

Day	Metabolic Pathways	
	Male Mussels	Female Mussels
1	Phenylalanine † (1) Phenylalanine, tyrosine & tryptophan biosynthesis † (1)	Valine, leucine & isoleucine degradation * (2) Valine, leucine & isoleucine biosynthesis * (2) **Glycerophospholipid** * (3) **Pyrimidine** * (4) **Pentose phosphate pathway** * Glycerolipid † Ether lipid † (5) Pyruvate † (6) Glycolysis/gluconeogenesis † (7)
3	*Steroid hormone biosynthesis* * Vitamin B6 * Phenylalanine, tyrosine & tryptophan biosynthesis * (1) Phenylalanine * (1) Glycine, serine & threonine † (8) Aminoacyl-tRNA biosynthesis † (9) Tryptophan † (1) Ether lipid † (5) Biotin † (10)	Phenylalanine * (1) Phenylalanine, Tyrosine & Tryptophan biosynthesis * (1) Aminoacyl-tRNA biosynthesis * (9) Tyrosine * (1) *D*-arginine & *D*-ornithine * (11) Glycine, serine & threonine * (8) **Cysteine & methionine** * Biotin * (10) **Primary bile acid biosynthesis †** **Glycerophospholipid † (3)** **Pyrimidine** † (4) Ether lipid † (5)
7	Glycolysis/Gluconeogenesis * (7) Pyruvate * (6) beta-Alanine * (12) Alanine, aspartate & glutamate * (12) *Propanoate* * Nicotinate & nicotinamide * Pantothenate & CoA biosynthesis * Citrate cycle * Butanoate * Histidine † Aminoacyl-tRNA biosynthesis † (9) Arginine biosynthesis † (11) Valine, leucine & isoleucine degradation † (2) *Amino sugar & nucleotide sugar* †	**Purine** * Glutathione * Valine, leucine & isoleucine degradation * (2) Valine, leucine & isoleucine biosynthesis * (2) **Nitrogen** * D-glutamine & *D*-glutamate * (12) Aminoacyl-tRNA biosynthesis * (9) Glycolysis/gluconeogenesis † (7) Pyruvate † (6) Arginine & proline † (11) Biotin † (10)

**Table 3:** Number of mussels in each exposure condition (control or exposed) according to sampling time and sex.

Day	Male Mussels		Female Mussels		Undifferentiated		Dead	
	Controls	Exposed	Controls	Exposed	Controls	Exposed	Controls	Exposed
1	13	7 (11)	9	14	2	/	1	/
3	8	11 (12)	15	13	1	/	1	/
7	11	15	12	6 (7)	/	3	2	/

## Data Availability

The data presented in this study are available in article and supplementary materials.

## Supplementary Materials

The following supporting information can be downloaded at: https://www.mdpi.com/article/10.3390/metabo12030197/s1, Table S1: Complete table of modulated metabolites in digestive glands of male and female mussels after 1, 3, and 7 days of exposure to VLF sorted by their metabolic pathway, Figure S1: Quantitative enrichment analysis for male mussels exposed to 10 µg/L of venlafaxine after 1 day (A), 3 days (B), and 7 days (C), Figure S2: Quantitative enrichment analysis for female mussels exposed to 10 µg/L of venlafaxine after 1 day (A), 3 days (B), and 7 days (C).
