# Supplementary material for: Early Biological Modulations Resulting from 1-Week Venlafaxine Exposure of Marine Mussels Mytilus galloprovincialis Determined by a Metabolomic Approach
Source: Metabolites. 2022 Feb 22;12(3):197. doi: 10.3390/metabo12030197 (PMC8949932; doi:10.3390/metabo12030197)

Supplementary data

***Table S1**: Complete table of modulated metabolites in digestive glands of male and female mussels after 1, 3 and 7 days of exposure to VLF sorted by their metabolic pathway (†: metabolites present in several pathways). Difference amplitudes are expressed in percentages. Bold values are for significant modulations (p <0.05), other black values represent a trend of modulation (p < 0.1) and grey values are for modulations with a p-value > 0.1. Slashes represent signals with a difference amplitude < 20% and are considered as not modulated. ND: Not Detected. Some metabolites from different pathways were not able to differentiate and a number in brackets was attributed to them.*

Metabolism Pathway	Metabolite	Rt (min)	Adduct	Calculated mass	Observed mass	Δm/z (ppm)	RSD QC_M	M1 Diff Amp	M1 pvalue	M3 Diff Amp	M3 pvalue	M7 Diff Amp	M7 pvalue	RSD QC_F	F1 Diff Amp	F1 pvalue	F3 Diff Amp	F3 pvalue	RSD QC	F7 Diff Amp	F7 pvalue	Annotation level
Acetylcholine synthesis	Glycerophosphorylcholine	1,53	M+H+	258,11010	258,10972	1,5	13,42	/	> 0.1	**/**	< 0.05	/	> 0.1	7,82	↗+29	< 0.1	↗+25	< 0.1	4,00	/	> 0.1	1
	Propionylcholine	4,46	M+	160,13375	160,13307	4,3	4,78	**↗+90**	< 0.05	↘-25	> 0.1	/	> 0.1	8,36	/	> 0.1	/	> 0.1	5,17	↗+20	> 0.1	2
Alanine, aspartate and glutamate Metabolism	Acetic acid† (1)	1,22	M+FA-H-	105,01933	105,01920	1,2	40,84	/	> 0.1	/	> 0.1	/	> 0.1	5,52	**/**	< 0.05	/	> 0.1	2,71	/	> 0.1	1
	Aspartylphenylalanine†	11,03	M+H+	281,11320	281,11302	0,6	5,87	**↗+70**	< 0.05	**↘-39**	< 0.05	**↘-28**	< 0.05	13,03	**↘-38**	< 0.05	**↘-27**	< 0.05	11,46	↘-31	> 0.1	2
	Citric acid†	1,43	M-H-	191,01973	191,01947	1,3	6,52	↘-43	> 0.1	↗+26	> 0.1	/	> 0.1	4,63	↗+104	> 0.1	↗+44	> 0.1	37,04	/	> 0.1	1
	Fumaric acid†	1,65	M-H-	115,00368	115,00356	1,1	62,36	/	> 0.1	/	> 0.1	/	> 0.1	3,66	/	< 0.1	/	> 0.1	89,01	/	> 0.1	1
	Gamma-l-glutamyl-l-leucine	9,63	M+H+	261,14450	261,14405	1,7	5,65	/	> 0.1	/	> 0.1	/	> 0.1	9,50	**↘-33**	< 0.05	↘-23	< 0.1	7,56	↗+26	> 0.1	1
	Glucosamine-6-phosphate†	0,97	M-H-	258,03843	258,03795	1,9	4,57	/	> 0.1	/	> 0.1	/	> 0.1	2,46	/	> 0.1	/	> 0.1	3,27	/	> 0.1	1
	Glyoxylic acid†	1,25	M-H-	72,99312	72,99286	3,5	43,46	**/**	< 0.05	/	> 0.1	/	> 0.1	3,90	/	< 0.1	/	> 0.1	2,55	/	> 0.1	1
	Isovalerylalanine	9,44	M+H+	174,11247	174,11235	0,7	7,99	/	> 0.1	/	> 0.1	/	< 0.1	8,53	↘-34	< 0.05	**↘-31**	< 0.05	6,34	/	< 0.1	2
	L-Alanine† (2)	1,45	M+H+	90,05495	90,05490	0,6	3,24	/	> 0.1	↘-22	> 0.1	/	> 0.1	10,48	/	> 0.1	/	> 0.1	44,32	**/**	< 0.05	1
	L-aspartic acid†	1,27	M-H-	132,03023	132,02999	1,8	18,20	/	> 0.1	/	> 0.1	**/**	< 0.05	1,19	/	> 0.1	/	> 0.1	3,38	/	> 0.1	1
	L-citrulline†	1,28	M-H-	174,08842	174,08694	8,5	ND	ND	ND	ND	ND	ND	ND	61,54	/	> 0.1	/	> 0.1	ND	ND	ND	1
	L-Glutamic acid†	1,36	M-H-	146,04588	146,04563	1,7	19,06	/	> 0.1	/	> 0.1	/	> 0.1	1,94	/	> 0.1	/	> 0.1	2,83	/	> 0.1	1
	Malonic acid† (3)	1,52	M-H-	103,00368	103,00355	1,3	6,72	/	> 0.1	/	> 0.1	/	> 0.1	14,66	↗+98	> 0.1	↗+35	> 0.1	9,81	↘-24	> 0.1	1
	N-acetyl-L-aspartic acid	1,48	M-H-	174,04080	174,04055	1,4	4,65	↘-33	< 0.1	/	> 0.1	↗+25	> 0.1	4,79	/	> 0.1	↗+40	< 0.1	9,45	↘-40	< 0.1	1
	N-Acetyl-L-glutamic acid	1,85	M-H-	188,05645	188,05609	1,9	24,42	/	> 0.1	/	> 0.1	/	> 0.1	13,39	**↘-44**	< 0.05	/	> 0.1	9,17	/	> 0.1	1
	Succinic acid†	1,89	M-H-	117,01933	117,01914	1,6	4,98	↘-25	> 0.1	/	> 0.1	↗+85	< 0.1	2,51	↘-28	> 0.1	/	> 0.1	4,26	↗+24	> 0.1	1
Amino Sugar Metabolism	Acetic acid† (1)	1,22	M+FA-H-	105,01933	105,01920	1,2	40,84	/	> 0.1	/	> 0.1	/	> 0.1	5,52	**/**	< 0.05	/	> 0.1	2,71	/	> 0.1	1
	Glucosamine	2,07	M+H+	180,08665	180,08640	1,4	25,60	/	> 0.1	/	> 0.1	**↘-37**	< 0.05	16,32	/	> 0.1	/	> 0.1	18,44	↘-28	< 0.1	1
	Glucosamine-6-phosphate†	0,97	M-H-	258,03843	258,03795	1,9	4,57	/	> 0.1	/	> 0.1	/	> 0.1	2,46	/	> 0.1	/	> 0.1	3,27	/	> 0.1	1
	N-Acetyl-D-glucosamine or N-Acetylmannosamine	1,21	M+FA-H-	266,08814	266,08776	1,4	8,38	/	> 0.1	/	> 0.1	**↗+30**	< 0.05	10,46	/	> 0.1	/	> 0.1	1,59	↗+23	> 0.1	1
Arginine and Proline Metabolism and derivatives	2-Oxoarginine	1,92	M-H20-H-	154,06165	154,06192	-1,7	6,87	/	> 0.1	/	> 0.1	/	> 0.1	9,72	/	> 0.1	**↘-48**	< 0.05	27,51	/	> 0.1	2
	4-(Glutamylamino) butanoate	3,13	M+H+	233,11320	233,11304	0,7	20,30	/	> 0.1	/	> 0.1	/	> 0.1	6,53	/	< 0.1	**↘-31**	< 0.05	24,74	**↘-38**	< 0.05	2
	4-hydroxyproline† (4)	1,22	M+H+	132,06552	132,06536	1,2	39,47	**/**	< 0.05	/	> 0.1	/	> 0.1	8,48	/	> 0.1	↗+28	> 0.1	9,59	/	> 0.1	1
	5-aminovaleric acid†	3,07	M+H+	118,08625	118,08603	1,9	27,78	/	> 0.1	/	> 0.1	/	> 0.1	24,76	/	> 0.1	/	> 0.1	34,31	/	> 0.1	1
	Creatinine	13,07	2M+H+	227,12510	227,12511	-0,1	6,88	**↘-32**	< 0.05	/	> 0.1	/	> 0.1	16,98	/	> 0.1	/	> 0.1	42,97	/	> 0.1	1
	Fumaric acid†	1,65	M-H-	115,00368	115,00356	1,1	62,36	/	> 0.1	/	> 0.1	/	> 0.1	3,66	/	< 0.1	/	> 0.1	89,01	/	> 0.1	1
	Gamma-glutamyl-L-putrescine	3,94	M-H2O+H+	200,13880	200,13936	-2,8	19,66	↗+23	> 0.1	↘-44	< 0.1	/	> 0.1	21,84	/	> 0.1	↘-37	> 0.1	ND	ND	ND	2
	Glyoxylic acid†	1,25	M-H-	72,99312	72,99286	3,5	43,46	**/**	< 0.05	/	> 0.1	/	> 0.1	3,90	/	< 0.1	/	> 0.1	2,55	/	> 0.1	1
	L-4-Hydroxyglutamate semialdehyde	1,56	M-H20-H-	128,03477	128,03516	-3,1	12,22	/	> 0.1	↘-22	> 0.1	/	> 0.1	4,23	/	> 0.1	↗+34	> 0.1	5,77	**↘-36**	< 0.05	2
	L-Arginine†	2,63	M+H+	175,11895	175,11873	1,3	17,58	/	> 0.1	/	> 0.1	/	> 0.1	22,52	↗+39	> 0.1	/	< 0.1	6,56	↘-38	< 0.1	1
	L-Aspartate-semialdehyde†	1,44	M-H-	116,03532	116,03517	1,3	5,04	↘-25	> 0.1	/	> 0.1	↗+22	> 0.1	3,23	/	> 0.1	**↗+28**	< 0.05	4,14	/	> 0.1	2
	L-aspartic acid†	1,27	M-H-	132,03023	132,02999	1,8	18,20	/	> 0.1	/	> 0.1	**/**	< 0.05	1,19	/	> 0.1	/	> 0.1	3,38	/	> 0.1	1
	L-citrulline†	1,28	M-H-	174,08842	174,08694	8,5	ND	ND	ND	ND	ND	ND	ND	61,54	/	> 0.1	/	> 0.1	ND	ND	ND	1
	L-Glutamic acid†	1,36	M-H-	146,04588	146,04563	1,7	19,06	/	> 0.1	/	> 0.1	/	> 0.1	1,94	/	> 0.1	/	> 0.1	2,83	/	> 0.1	1
	L-Glutamine†	1,26	M+H+	147,07642	147,07623	1,3	10,01	/	> 0.1	↘-23	> 0.1	**/**	< 0.05	5,26	/	> 0.1	**↘-38**	< 0.05	9,98	**↘-33**	< 0.05	1
	L-Ornithine†	2,35	M+H+	133,09715	133,09695	1,5	5,46	/	> 0.1	/	> 0.1	/	> 0.1	54,70	**/**	< 0.05	**/**	< 0.05	6,51	↘-32	> 0.1	1
	L-Proline	1,42	M+H+	116,07060	116,07057	0,3	4,09	/	> 0.1	/	> 0.1	/	> 0.1	8,77	**/**	< 0.05	/	> 0.1	2,50	/	> 0.1	1
	N-acetylproline	3,01	M-H-	156,06662	156,06628	2,2	9,59	/	> 0.1	↘-20	> 0.1	↗+58	> 0.1	7,08	/	> 0.1	/	> 0.1	6,06	/	> 0.1	1
	N4-Acetylaminobutanal (5)	2,04	M+H+	130,08625	130,08620	0,4	8,24	↗+27	> 0.1	/	> 0.1	↗+26	> 0.1	11,48	↘-37	> 0.1	↘-22	> 0.1	6,41	↗+28	> 0.1	2

Metabolism Pathway	Metabolite	Rt (min)	Adduct	Calculated mass	Observed mass	Δm/z (ppm)	RSD QC_M	M1 Diff Amp	M1 pvalue	M3 Diff Amp	M3 pvalue	M7 Diff Amp	M7 pvalue	RSD QC_F	F1 Diff Amp	F1 pvalue	F3 Diff Amp	F3 pvalue	RSD QC	F7 Diff Amp	F7 pvalue	Annotation level
Arginine and Proline Metabolism and derivatives	Proline betaine	1,49	M+H+	144,10190	144,10174	1,1	3,72	/	> 0.1	**/**	< 0.05	/	> 0.1	4,32	/	> 0.1	/	> 0.1	4,67	↘-26	> 0.1	1
	Putrescine†	5,43	M+H+	89,10732	89,10718	1,6	24,40	/	> 0.1	/	> 0.1	/	> 0.1	30,59	/	> 0.1	/	> 0.1	27,43	/	> 0.1	1
	Sarcosine† (2)	1,45	M+H+	90,05495	90,05490	0,6	3,24	/	> 0.1	↘-22	> 0.1	/	> 0.1	10,48	/	> 0.1	/	> 0.1	44,32	**/**	< 0.05	1
	Succinic acid†	1,89	M-H-	117,01933	117,01914	1,6	4,98	↘-25	> 0.1	/	> 0.1	↗+85	< 0.1	2,51	↘-28	> 0.1	/	> 0.1	4,26	↗+24	> 0.1	1
	Symmetric dimethylarginine	3,45	M+H+	203,15025	203,15016	0,4	13,70	/	> 0.1	↘-21	< 0.1	↘-27	> 0.1	6,80	/	< 0.1	**↘-46**	< 0.05	4,45	**↘-47**	< 0.05	2
beta-Alanine Metabolism	Hydroxypropionic acid†	1,55	M-H-	89,02442	89,02440	0,2	7,75	/	> 0.1	/	> 0.1	**↗+68**	< 0.05	3,98	/	> 0.1	**/**	< 0.05	6,90	↘-26	> 0.1	1
	L-aspartic acid†	1,27	M-H-	132,03023	132,02999	1,8	18,20	/	> 0.1	/	> 0.1	**/**	< 0.05	1,19	/	> 0.1	/	> 0.1	3,38	/	> 0.1	1
	L-Histidine†	2,23	M+H+	156,07675	156,07670	0,3	4,58	/	> 0.1	/	> 0.1	/	< 0.1	40,78	/	> 0.1	/	> 0.1	5,61	/	> 0.1	1
	N-Acetyl-beta-alanine (4)	1,77	M-H-	130,05097	130,05079	1,4	5,91	/	> 0.1	/	> 0.1	/	> 0.1	1,91	**/**	< 0.05	/	< 0.1	10,49	/	> 0.1	1
	Pantothenic acid	2,64	M-H-	218,10340	218,10307	1,5	8,70	/	> 0.1	↘-20	> 0.1	/	> 0.1	3,37	/	> 0.1	/	> 0.1	4,07	↗+24	> 0.1	1
	Uracil†	1,52	M-H-	111,02000	111,01988	1,1	6,25	/	> 0.1	/	> 0.1	/	> 0.1	16,21	**↘-40**	< 0.05	/	> 0.1	5,90	/	> 0.1	1
Betaine Metabolism	Betaine†	1,33	M+H+	118,08625	118,08605	1,7	4,03	/	> 0.1	/	> 0.1	/	> 0.1	9,17	/	> 0.1	**/**	< 0.05	3,63	/	> 0.1	1
	Choline†	2,51	M+	104,10754	104,10685	6,6	4,35	/	> 0.1	/	> 0.1	/	> 0.1	11,16	/	< 0.1	/	> 0.1	2,55	/	> 0.1	1
	L-Lysine†	2,50	M+H+	147,11280	147,11261	1,3	5,22	/	> 0.1	↘-33	> 0.1	/	> 0.1	12,04	↗+39	> 0.1	**↘-57**	< 0.05	4,00	↘-38	> 0.1	1
	Pimelic acid	8,54	M-H-	159,06628	159,06601	1,7	1,98	↗+61	> 0.1	↘-25	> 0.1	/	> 0.1	3,23	↗+47	< 0.1	↗+21	> 0.1	2,51	/	> 0.1	1
Citrate cycle	Citric acid†	1,43	M-H-	191,01973	191,01947	1,3	6,52	↘-43	> 0.1	↗+26	> 0.1	/	> 0.1	4,63	↗+104	> 0.1	↗+44	> 0.1	37,04	/	> 0.1	1
	Fumaric acid†	1,65	M-H-	115,00368	115,00356	1,1	62,36	/	> 0.1	/	> 0.1	/	> 0.1	3,66	/	< 0.1	/	> 0.1	89,01	/	> 0.1	1
	L-malic acid†	1,33	M-H-	133,01425	133,01402	1,7	3,82	/	> 0.1	/	> 0.1	/	> 0.1	3,09	/	< 0.05	/	> 0.1	4,74	**/**	< 0.05	1
Collagen Synthesis	4-hydroxyproline† (4)	1,22	M+H+	132,06552	132,06536	1,2	39,47	**/**	< 0.05	/	> 0.1	/	> 0.1	8,48	/	> 0.1	↗+28	> 0.1	9,59	/	> 0.1	1
	Glycylproline	3,25	M+H+	173,09207	173,09164	2,5	21,86	/	> 0.1	**/**	< 0.05	/	> 0.1	31,78	/	> 0.1	/	> 0.1	8,34	/	> 0.1	1
Cysteine and methionine Metabolism	2-Hydroxyphenethylamine	7,81	M-H2O+H+	120,08023	120,08059	-3,0	2,68	↗+30	< 0.05	↘-30	< 0.05	/	< 0.1	3,27	↘-22	< 0.05	**↘-46**	< 0.05	4,81	↘-27	< 0.1	2
	Betaine†	1,33	M+H+	118,08625	118,08605	1,7	4,03	/	> 0.1	/	> 0.1	/	> 0.1	9,17	/	> 0.1	**/**	< 0.05	3,63	/	> 0.1	1
	Choline†	2,51	M+	104,10754	104,10685	6,6	4,35	/	> 0.1	/	> 0.1	/	> 0.1	11,16	/	< 0.1	/	> 0.1	2,55	/	> 0.1	1
	L-Alanine† (2)	1,45	M+H+	90,05495	90,05490	0,6	3,24	/	> 0.1	↘-22	> 0.1	/	> 0.1	10,48	/	> 0.1	/	> 0.1	44,32	**/**	< 0.05	1
	L-alpha-Aminobutyric acid	1,71	M+H+	104,07060	104,07049	1,1	41,46	/	> 0.1	/	> 0.1	/	> 0.1	69,37	/	> 0.1	/	> 0.1	54,30	↘-65	> 0.1	1
	L-Aspartate-semialdehyde†	1,44	M-H-	116,03532	116,03517	1,3	5,04	↘-25	> 0.1	/	> 0.1	↗+22	> 0.1	3,23	/	> 0.1	**↗+28**	< 0.05	4,14	/	> 0.1	2
	L-aspartic acid†	1,27	M-H-	132,03023	132,02999	1,8	18,20	/	> 0.1	/	> 0.1	**/**	< 0.05	1,19	/	> 0.1	/	> 0.1	3,38	/	> 0.1	1
	L-Methionine†	2,57	M+H+	150,05833	150,05769	4,2	37,34	**/**	< 0.05	/	> 0.1	/	> 0.1	9,79	**↘-42**	< 0.05	**↘-63**	< 0.05	12,00	↘-40	> 0.1	1
	L-serine†	1,31	M-H-	104,03532	104,03522	1,0	6,18	/	> 0.1	/	> 0.1	/	> 0.1	5,64	/	> 0.1	↘-22	> 0.1	8,36	/	> 0.1	1
	Putrescine†	5,43	M+H+	89,10732	89,10718	1,6	24,40	/	> 0.1	/	> 0.1	/	> 0.1	30,59	/	> 0.1	/	> 0.1	27,43	/	> 0.1	1
Glutathione Metabolism	5-l-glutamyl-l-alanine	1,69	M+H+	219,09755	219,09747	0,4	16,97	/	> 0.1	/	> 0.1	/	> 0.1	17,90	**↘-33**	< 0.05	/	> 0.1	ND	ND	ND	1
	Cysteine glutathione disulfide	1,30	M-H-	425,08063	425,07952	2,6	ND	ND	ND	ND	ND	ND	ND	27,02	↗+94	< 0.05	**↗+71**	< 0.05	ND	ND	ND	1
	Glycine†	1,34	M+H+	76,03930	76,03914	2,2	16,67	↘-29	> 0.1	/	> 0.1	↘-21	< 0.1	49,04	/	> 0.1	**↗+85**	< 0.05	43,21	↘-36	> 0.1	1
	L-Glutamic acid†	1,36	M-H-	146,04588	146,04563	1,7	19,06	/	> 0.1	/	> 0.1	/	> 0.1	1,94	/	> 0.1	/	> 0.1	2,83	/	> 0.1	1
	L-Ornithine†	2,35	M+H+	133,09715	133,09695	1,5	5,46	/	> 0.1	/	> 0.1	/	> 0.1	54,70	**/**	< 0.05	**/**	< 0.05	6,51	↘-32	> 0.1	1
	Oxidized glutathione	2,17	M-H-	611,14469	611,14429	0,6	18,02	/	> 0.1	↘-31	> 0.1	/	> 0.1	3,81	↗+59	> 0.1	/	> 0.1	11,82	/	> 0.1	1
	Putrescine†	5,43	M+H+	89,10732	89,10718	1,6	24,40	/	> 0.1	/	> 0.1	/	> 0.1	30,59	/	> 0.1	/	> 0.1	27,43	/	> 0.1	1
	Pyroglutamic acid	1,56	M-H-	128,03532	128,03517	1,1	12,22	/	> 0.1	↘-22	> 0.1	/	> 0.1	4,23	/	> 0.1	↗+34	> 0.1	5,90	**↘-33**	< 0.05	1
Glycine, serine and threonine Metabolism	5-aminolevulinic acid (4)	1,22	M+H+	132,06552	132,06536	1,2	39,47	**/**	< 0.05	/	> 0.1	/	> 0.1	8,48	/	> 0.1	↗+28	> 0.1	9,59	/	> 0.1	1
	Aminoacetone	1,33	M+FA-H-	118,05096	118,05084	1,1	3,18	/	> 0.1	/	> 0.1	/	> 0.1	4,40	/	> 0.1	**↘-25**	< 0.05	2,94	/	> 0.1	2
	Betaine†	1,33	M+H+	118,08625	118,08605	1,7	4,03	/	> 0.1	/	> 0.1	/	> 0.1	9,17	/	> 0.1	**/**	< 0.05	3,63	/	> 0.1	1
	Choline†	2,51	M+	104,10754	104,10685	6,6	4,35	/	> 0.1	/	> 0.1	/	> 0.1	11,16	/	< 0.1	/	> 0.1	2,55	/	> 0.1	1
	DL-2-hydroxybutyric acid	2,09	M-H-	103,04007	103,03995	1,1	19,17	↗+23	> 0.1	/	> 0.1	/	> 0.1	4,99	**↗+43**	< 0.05	/	> 0.1	19,70	/	> 0.1	1
	Glyceric acid† (1)	1,24	M-H-	105,01933	105,01917	1,5	2,66	/	> 0.1	/	> 0.1	/	> 0.1	4,38	**/**	< 0.05	/	> 0.1	0,85	/	> 0.1	1
	Glycine†	1,34	M+H+	76,03930	76,03914	2,2	16,67	↘-29	> 0.1	/	> 0.1	↘-21	< 0.1	49,04	/	> 0.1	**↗+85**	< 0.05	43,21	↘-36	> 0.1	1
	Glyoxylic acid†	1,25	M-H-	72,99312	72,99286	3,5	43,46	**/**	< 0.05	/	> 0.1	/	> 0.1	3,90	/	< 0.1	/	> 0.1	2,55	/	> 0.1	1
	Hydroxypyruvic acid (3)	1,52	M-H-	103,00368	103,00355	1,3	6,28	/	> 0.1	/	> 0.1	/	> 0.1	18,51	↗+68	> 0.1	**↗+191**	< 0.05	10,22	↘-22	> 0.1	1
	L-allothreonine or L-threonine	1,33	M-H-	118,05097	118,05084	1,1	3,18	/	> 0.1	/	> 0.1	/	> 0.1	4,40	/	> 0.1	**↘-25**	< 0.05	101,41	↗+47	> 0.1	1
	L-Arginine†	2,63	M+H+	175,11895	175,11873	1,3	17,58	/	> 0.1	/	> 0.1	/	> 0.1	22,52	↗+39	> 0.1	/	< 0.1	6,56	↘-38	< 0.1	1
	L-Aspartate-semialdehyde†	1,44	M-H-	116,03532	116,03517	1,3	5,04	↘-25	> 0.1	/	> 0.1	↗+22	> 0.1	3,23	/	> 0.1	**↗+28**	< 0.05	4,14	/	> 0.1	2
	L-aspartic acid†	1,27	M-H-	132,03023	132,02999	1,8	18,20	/	> 0.1	/	> 0.1	**/**	< 0.05	1,19	/	> 0.1	/	> 0.1	3,38	/	> 0.1	1

Metabolism Pathway	Metabolite	Rt (min)	Adduct	Calculated mass	Observed mass	Δm/z (ppm)	RSD QC_M	M1		M3		M7		RSD QC_F	F1		F3		RSD QC	F7		Annotation level
								Diff Amp	pvalue	Diff Amp	pvalue	Diff Amp	pvalue		Diff Amp	pvalue	Diff Amp	pvalue		Diff Amp	pvalue	
Glycine, serine and threonine Metabolism	L-Homoserine	1,38	M+H+	120,06552	120,06537	1,2	16,80	↘-26	< 0.1	↘-20	> 0.1	/	> 0.1	27,68	/	> 0.1	/	> 0.1	6,97	/	> 0.1	1
	L-Methionine†	2,57	M+H+	150,05833	150,05769	4,2	37,34	/	< 0.05	/	> 0.1	/	> 0.1	9,79	↘-42	< 0.05	↘-63	< 0.05	12,00	↘-40	> 0.1	1
	L-serine†	1,31	M-H-	104,03532	104,03522	1,0	6,18	/	> 0.1	/	> 0.1	/	> 0.1	5,64	/	> 0.1	↘-22	> 0.1	8,36	/	> 0.1	1
	L-Tryptophan†	11,58	M+H+	205,09715	205,09700	0,7	6,00	/	> 0.1	↘-29	< 0.05	/	> 0.1	7,93	/	< 0.05	↘-46	< 0.05	26,28	/	> 0.1	1
	N-Acetyl-L-threonine	1,50	M-H-	160,06153	160,06128	1,6	10,64	↘-24	> 0.1	/	> 0.1	↗+32	< 0.1	13,62	↘-26	< 0.05	↗+27	< 0.05	21,75	/	> 0.1	1
	Sarcosine† (2)	1,45	M+H+	90,05495	90,05490	0,6	3,24	/	> 0.1	↘-22	> 0.1	/	> 0.1	10,48	/	> 0.1	/	> 0.1	44,32	/	< 0.05	1
Histidine Metabolism	3'-AMP† or Adenosine monophosphate†	1,21	M+H+	348,07036	348,06994	1,2	22,88	/	> 0.1	↘-37	> 0.1	/	> 0.1	17,48	↗+472	> 0.1	/	> 0.1	16,86	/	> 0.1	1
	Carnosine	3,56	M+H+	227,11387	227,11384	0,1	ND	ND	ND	ND	ND	ND	ND	35,44	/	> 0.1	↘-51	< 0.05	ND	ND	ND	1
	Histamine	5,84	M+H+	112,08692	112,08671	1,9	60,63	↘-75	< 0.05	↗+188	< 0.05	↗+108	> 0.1	13,54	↘-36	< 0.1	↘-26	> 0.1	39,81	↗+115	> 0.1	1
	L-aspartic acid†	1,27	M-H-	132,03023	132,02999	1,8	18,20	/	> 0.1	/	> 0.1	/	< 0.05	1,19	/	> 0.1	/	> 0.1	3,38	/	> 0.1	1
	L-Glutamic acid†	1,36	M-H-	146,04588	146,04563	1,7	19,06	/	> 0.1	/	> 0.1	/	> 0.1	1,94	/	> 0.1	/	> 0.1	2,83	/	> 0.1	1
	L-Histidine†	2,23	M+H+	156,07675	156,07670	0,3	4,58	/	> 0.1	/	> 0.1	/	< 0.1	40,78	/	> 0.1	/	> 0.1	5,61	/	> 0.1	1
	Methylimidazoleacetic acid	3,10	M+H+	141,06585	141,06572	0,9	24,65	/	> 0.1	/	> 0.1	/	> 0.1	2,79	/	> 0.1	↘-21	< 0.1	ND	ND	ND	1
Lysine biosynthesis or degradation	5-aminovaleric acid†	3,07	M+H+	118,08625	118,08603	1,9	27,78	/	> 0.1	/	> 0.1	/	> 0.1	24,76	/	> 0.1	/	> 0.1	34,31	/	> 0.1	1
	Aminoadipic acid	1,59	M+H+	162,07608	162,07598	0,7	37,41	/	> 0.1	/	> 0.1	/	> 0.1	107,20	↗+189	< 0.1	/	> 0.1	7,24	/	> 0.1	1
	Glutaric acid	2,48	M-H-	131,03498	131,03474	1,9	5,23	↗+34	> 0.1	/	> 0.1	/	> 0.1	3,12	↗+41	< 0.05	↗+27	> 0.1	4,06	/	> 0.1	1
	Glycine†	1,34	M+H+	76,03930	76,03914	2,2	16,67	↘-29	> 0.1	/	> 0.1	↘-21	< 0.1	49,04	/	> 0.1	↗+85	< 0.05	43,21	↘-36	> 0.1	1
	L-Aspartate-semialdehyde†	1,44	M-H-	116,03532	116,03517	1,3	5,04	↘-25	> 0.1	/	> 0.1	↗+22	> 0.1	3,23	/	> 0.1	↗+28	< 0.05	4,14	/	> 0.1	2
	L-aspartic acid†	1,27	M-H-	132,03023	132,02999	1,8	18,20	/	> 0.1	/	> 0.1	/	< 0.05	1,19	/	> 0.1	/	> 0.1	3,38	/	> 0.1	1
	L-Lysine†	2,50	M+H+	147,11280	147,11261	1,3	5,22	/	> 0.1	↘-33	> 0.1	/	> 0.1	12,04	↗+39	> 0.1	↘-57	< 0.05	4,00	↘-38	> 0.1	1
	N-Succinyl-2-amino-6-ketopimelate	2,24	M+H+	290,08704	290,08578	4,4	34,31	/	> 0.1	↘-63	> 0.1	/	> 0.1	19,31	↘-37	< 0.1	↘-50	< 0.05	17,40	/	> 0.1	2
	N6-Acetyl-L-lysine	1,89	M+H+	189,12337	189,12302	1,8	32,68	/	> 0.1	/	> 0.1	↗+108	> 0.1	29,58	/	> 0.1	/	< 0.05	7,80	/	> 0.1	1
	Oxoadipic acid†	1,63	M-H-	159,02990	159,02996	-0,4	51,01	/	< 0.05	/	> 0.1	ND	ND	ND	ND	ND	ND	ND	ND	ND	ND	1
	Pipecolic acid (5)	2,04	M+H+	130,08625	130,08620	0,4	8,24	↗+27	> 0.1	/	> 0.1	↗+26	> 0.1	11,48	↘-37	> 0.1	↘-22	> 0.1	11,55	↗+38	< 0.1	1
Nicotinate and Nicotinamide Metabolism	L-aspartic acid†	1,27	M-H-	132,03023	132,02999	1,8	18,20	/	> 0.1	/	> 0.1	/	< 0.05	1,19	/	> 0.1	/	> 0.1	3,38	/	> 0.1	1
	Niacinamide	2,46	M+H+	123,05529	123,05511	1,5	10,36	↗+71	> 0.1	↘-49	< 0.1	/	> 0.1	33,47	↗+72	< 0.1	/	> 0.1	4,38	/	> 0.1	1
	Nicotinamide N-oxide	3,46	M+H+	139,05020	139,05005	1,1	8,02	↗+32	> 0.1	/	> 0.1	↗+256	< 0.05	8,94	/	> 0.1	/	> 0.1	8,68	↘-41	< 0.05	2
	Nicotinamide riboside	14,29	M+	255,09810	255,09892	-3,2	12,38	/	< 0.05	/	< 0.05	/	> 0.1	13,07	/	> 0.1	/	> 0.1	2,52	↘-35	> 0.1	1
	Nicotinic acid	1,85	M+H+	124,03930	124,03925	0,4	9,51	/	> 0.1	/	> 0.1	/	> 0.1	3,71	/	> 0.1	/	> 0.1	22,46	↘-46	> 0.1	1
	Nicotinuric acid	2,61	M-H-	179,04622	179,04597	1,4	ND	ND	ND	ND	ND	ND	ND	20,47	/	> 0.1	/	> 0.1	24,90	/	> 0.1	1
	Propionic acid	2,02	M-H-	73,02950	73,02933	2,4	45,91	↗+69	> 0.1	/	> 0.1	↗+36	> 0.1	3,67	↘-34	> 0.1	↗+22	> 0.1	3,84	↗+27	> 0.1	1
	Trigonelline	1,45	M+H+	138,05495	138,05480	1,1	7,20	/	> 0.1	↘-45	< 0.05	/	> 0.1	12,60	/	> 0.1	/	> 0.1	23,87	/	> 0.1	1
Pentose phosphate pathway	Deoxyribose	1,21	M+FA-H-	179,05611	179,05589	1,2	4,25	/	> 0.1	↗+25	> 0.1	↗+43	< 0.05	4,00	↘-25	< 0.1	/	> 0.1	7,30	/	> 0.1	1
	Gluconic acid	1,15	M-H-	195,05103	195,05090	0,7	4,87	/	< 0.1	/	> 0.1	↗+31	< 0.1	6,37	/	> 0.1	/	> 0.1	34,87	↘-65	> 0.1	1
	Glyceric acid† (1)	1,24	M-H-	105,01933	105,01917	1,5	2,66	/	> 0.1	/	> 0.1	/	> 0.1	4,38	/	< 0.05	/	> 0.1	0,85	/	> 0.1	1
	Ribose 1-phosphate†	2,93	M-H2O+H+	213,01532	213,01594	-2,9	23,48	↗+37	> 0.1	/	> 0.1	/	> 0.1	16,43	↗+86	< 0.05	↗+26	> 0.1	15,67	/	> 0.1	2
Phenylalanine Metabolism	2-Phenylacetamide	7,97	M+H+	136,07569	136,07553	1,2	10,47	↗+21	> 0.1	↘-22	< 0.1	/	> 0.1	60,23	/	> 0.1	/	< 0.05	52,78	/	> 0.1	1
	3-(2-Hydroxyphenyl)propanoic acid	11,30	M-H-	165,05572	165,05520	3,1	58,73	/	> 0.1	/	> 0.1	/	> 0.1	35,75	/	> 0.1	/	> 0.1	3,59	/	> 0.1	1
	3-(3-Hydroxyphenyl)propanoic acid	10,79	M-H-	165,05572	165,05529	2,6	30,39	/	> 0.1	/	> 0.1	/	> 0.1	19,96	↗+42	< 0.05	↗+21	> 0.1	25,98	↗+41	< 0.1	1
	3-Hydroxyphenylacetic acid†	9,69	M-H-	151,04007	151,03971	2,4	5,47	/	> 0.1	/	> 0.1	/	> 0.1	15,54	/	> 0.1	/	> 0.1	5,06	/	> 0.1	1
	Aspartylphenylalanine†	11,03	M+H+	281,11320	281,11302	0,6	5,87	↗+70	< 0.05	↘-39	< 0.05	↘-28	< 0.05	13,03	↘-38	< 0.05	↘-27	< 0.05	11,46	↘-31	> 0.1	2
	Fumaric acid†	1,65	M-H-	115,00368	115,00356	1,1	62,36	/	> 0.1	/	> 0.1	/	> 0.1	3,66	/	< 0.1	/	> 0.1	89,01	/	> 0.1	1
	L-3-phenyllactic acid	10,48	M-H-	165,05572	165,05522	3,0	3,24	/	> 0.1	↗+130	< 0.05	/	> 0.1	4,32	↘-55	< 0.05	/	> 0.1	4,38	↗+32	> 0.1	1
	L-Phenylalanine	7,81	M+H+	166,08625	166,08613	0,8	3,39	↗+31	< 0.05	↘-31	< 0.05	/	< 0.05	2,89	↘-24	< 0.05	↘-45	< 0.05	14,10	↘-51	> 0.1	1
	L-Tyrosine†	3,54	M+H+	182,08117	182,08109	0,4	3,48	/	> 0.1	↘-21	< 0.1	/	> 0.1	7,07	/	< 0.05	↘-37	< 0.05	7,35	/	> 0.1	1
	N-Acetyl-L-phenylalanine	10,66	M-H-	206,08227	206,08182	2,2	3,46	/	> 0.1	/	> 0.1	/	> 0.1	15,02	↘-33	< 0.05	↘-26	< 0.05	4,79	/	> 0.1	1
	Phenylacetylglutamine	8,55	M-H-	263,10373	263,10342	1,2	21,98	↘-43	> 0.1	↘-38	> 0.1	/	> 0.1	4,32	/	> 0.1	/	> 0.1	4,76	/	> 0.1	1
	Phenylacetylglycine	9,53	M-H-	192,06662	192,06639	1,2	4,68	↘-37	< 0.05	/	> 0.1	/	> 0.1	4,99	↘-29	> 0.1	/	> 0.1	2,33	/	> 0.1	1
	Phenylethylamine	13,93	M+H+	122,09643	122,09624	1,5	17,46	/	> 0.1	/	< 0.05	/	> 0.1	6,51	/	> 0.1	↘-41	< 0.05	8,09	/	> 0.1	1

Metabolism Pathway	Metabolite	Rt (min)	Adduct	Calculated mass	Observed mass	Δm/z (ppm)	RSD QC_M	M1 Diff Amp	M1 pvalue	M3 Diff Amp	M3 pvalue	M7 Diff Amp	M7 pvalue	RSD QC_F	F1 Diff Amp	F1 pvalue	F3 Diff Amp	F3 pvalue	RSD QC	F7 Diff Amp	F7 pvalue	Annotation level
Purine Metabolism	2-Hydroxyadenine	2,34	M+Na+	174,03863	174,03854	0,5	8,40	↗+22	> 0.1	↗+71	< 0.1	/	> 0.1	15,43	/	< 0.1	/	> 0.1	10,67	↗+24	> 0.1	2
	2'-Deoxyguanosine	2,27	M+H+	268,10403	268,10376	1,0	6,15	↗+45	< 0.05	↘-36	> 0.1	/	> 0.1	16,70	↘-27	> 0.1	↘-47	< 0.05	ND	ND	ND	1
	3-Methylxanthine	0,97	M+FA-H-	211,04728	211,04756	-1,3	2,58	/	> 0.1	/	> 0.1	/	> 0.1	12,40	/	> 0.1	↗+24	> 0.1	3,28	/	> 0.1	2
	3'-AMP† or Adenosine monophosphate†	1,21	M+H+	348,07036	348,06994	1,2	22,88	/	> 0.1	↘-37	> 0.1	/	> 0.1	17,48	↗+472	> 0.1	/	> 0.1	16,86	/	> 0.1	1
	8-Hydroxy-deoxyguanosine or Guanosine	1,92	M-H-	282,08439	282,08414	0,9	3,83	/	< 0.05	/	> 0.1	/	< 0.1	10,03	/	> 0.1	/	< 0.1	7,49	/	> 0.1	1
	Adenine	2,89	M-H-	134,04722	134,04703	1,4	1,97	/	> 0.1	/	> 0.1	/	> 0.1	10,28	/	> 0.1	/	> 0.1	5,67	/	> 0.1	1
	Adenosine	3,14	M+H+	268,10403	268,10386	0,6	7,97	↘-52	> 0.1	↘-28	> 0.1	/	> 0.1	10,89	↗+117	> 0.1	↘-25	> 0.1	5,77	↘-36	> 0.1	1
	ADP	1,06	M-H-	426,02214	426,02168	1,1	23,97	/	> 0.1	/	> 0.1	/	> 0.1	ND	ND	ND	ND	ND	ND	ND	ND	1
	Cyclic amp	1,51	M-H-	328,04524	328,04500	0,7	7,21	↗+85	< 0.05	/	> 0.1	/	> 0.1	15,32	↘-40	< 0.1	↘-27	> 0.1	2,55	↘-30	> 0.1	1
	Deoxyinosine	1,87	M-H-	251,07858	251,07830	1,1	14,42	↗+52	< 0.1	↘-45	> 0.1	↘-27	> 0.1	9,40	↘-32	< 0.1	↘-53	< 0.05	9,75	↘-29	> 0.1	1
	dGDP	1,20	M-H-	426,02214	426,01991	5,2	24,18	/	> 0.1	/	> 0.1	/	> 0.1	28,46	↗+676	> 0.1	/	> 0.1	ND	ND	ND	1
	FAPy-adenine	2,29	M+H+	154,07234	154,07281	-3,1	33,99	/	> 0.1	/	> 0.1	/	> 0.1	ND	ND	ND	ND	ND	19,03	↘-25	> 0.1	1
	Glycine†	1,34	M+H+	76,03930	76,03914	2,2	16,67	↘-29	> 0.1	/	> 0.1	↘-21	< 0.1	49,04	/	> 0.1	↗+85	< 0.05	43,21	↘-36	> 0.1	1
	Glyoxylic acid†	1,25	M-H-	72,99312	72,99286	3,5	43,46	/	< 0.05	/	> 0.1	/	> 0.1	3,90	/	< 0.1	/	> 0.1	2,55	/	> 0.1	1
	Guanine	2,33	M+H+	152,05669	152,05655	0,9	5,22	↗+25	< 0.1	/	> 0.1	/	> 0.1	16,63	/	> 0.1	/	> 0.1	12,53	/	> 0.1	1
	Guanosine	1,92	M+H+	284,09894	284,09883	0,4	13,44	/	> 0.1	/	> 0.1	/	> 0.1	7,86	/	> 0.1	/	< 0.05	2,18	↗+34	> 0.1	1
	Guanosine monophosphate	1,10	M-H-	362,05072	362,05005	1,9	ND	ND	ND	ND	ND	ND	ND	23,25	/	< 0.1	↘-34	> 0.1	ND	ND	ND	1
	Hypoxanthine	1,74	M+H+	137,04579	137,04570	0,6	11,71	/	> 0.1	/	> 0.1	/	> 0.1	11,14	/	> 0.1	/	> 0.1	9,88	/	> 0.1	1
	Inosine	1,77	M-H-	267,07349	267,07331	0,7	4,60	/	> 0.1	/	> 0.1	/	< 0.05	5,92	/	> 0.1	/	> 0.1	1,75	/	> 0.1	1
	Inosine-5'-monophosphate	1,18	M-H-	347,03982	347,03989	-0,2	27,40	/	> 0.1	/	> 0.1	↗+42	> 0.1	6,09	/	> 0.1	↘-46	< 0.05	ND	ND	ND	1
	Ribose 1-phosphate†	2,93	M-H2O+H+	213,01532	213,01594	-2,9	23,48	↗+37	> 0.1	/	> 0.1	/	> 0.1	16,43	↗+86	< 0.05	↗+26	> 0.1	15,67	/	> 0.1	2
	Succinyladenosine	3,60	M+H+	384,11499	384,11485	0,4	23,18	/	> 0.1	↗+56	> 0.1	/	> 0.1	20,49	↘-39	< 0.05	/	> 0.1	4,11	↘-33	> 0.1	2
	Xanthine	1,64	M-H-	151,02615	151,02592	1,5	10,66	/	> 0.1	↗+79	< 0.05	/	> 0.1	7,59	↘-31	< 0.1	/	> 0.1	3,98	/	> 0.1	1
Pyrimidine Metabolism	3-methyluridine	2,27	M+FA-H-	303,08339	303,08270	2,3	ND	ND	ND	ND	ND	ND	ND	35,89	/	> 0.1	/	> 0.1	ND	ND	ND	1
	5-Hydroxymethyluracil	1,51	M-H-	141,03057	141,03029	2,0	45,93	/	> 0.1	/	> 0.1	/	< 0.1	31,43	/	> 0.1	↗+29	> 0.1	12,22	/	> 0.1	1
	5-Methylcytosine	2,31	M-H-	124,05164	124,05143	1,7	23,02	↘-48	< 0.05	↗+59	< 0.1	↗+62	> 0.1	16,40	↗+21	> 0.1	/	> 0.1	2,32	/	> 0.1	2
	Cytidine	2,66	M+H+	244,09280	244,09251	1,2	5,92	/	> 0.1	/	> 0.1	/	< 0.05	12,66	/	< 0.1	/	> 0.1	10,18	/	> 0.1	1
	Cytosine	2,41	M+H+	112,05054	112,05035	1,7	7,47	/	> 0.1	↗+32	> 0.1	/	> 0.1	18,06	↘-21	< 0.1	/	> 0.1	8,08	/	> 0.1	1
	Deoxycytidine	3,10	M+H+	228,09788	228,09765	1,0	7,90	/	> 0.1	/	> 0.1	↘-24	> 0.1	15,20	↘-36	< 0.05	↘-42	< 0.05	11,61	/	> 0.1	1
	Deoxyuridine	1,85	M+FA-H-	273,07282	273,07239	1,6	18,56	/	> 0.1	/	> 0.1	↘-42	> 0.1	ND	ND	ND	ND	ND	3,89	↘-23	> 0.1	1
	Dihydrothymine	1,97	M+H+	129,06585	129,06568	1,3	15,34	↘-41	> 0.1	↘-24	> 0.1	↗+58	< 0.05	3,23	/	> 0.1	↘-27	< 0.05	14,75	/	> 0.1	1
	DL-Lactic Acid†	6,38	M-H-	89,02442	89,02438	0,4	8,10	/	> 0.1	/	> 0.1	↗+60	< 0.05	10,87	/	> 0.1	↗+23	< 0.05	5,37	↘-27	> 0.1	1
	Hydroxypropionic acid†	1,55	M-H-	89,02442	89,02440	0,2	7,75	/	> 0.1	/	> 0.1	↗+68	< 0.05	3,98	/	> 0.1	/	< 0.05	6,90	↘-26	> 0.1	1
	L-Glutamine†	1,26	M+H+	147,07642	147,07623	1,3	10,01	/	> 0.1	↘-23	> 0.1	/	< 0.05	5,26	/	> 0.1	↘-38	< 0.05	9,98	↘-33	< 0.05	1
	Malonic acid† (3)	1,52	M-H-	103,00368	103,00355	1,3	6,72	/	> 0.1	/	> 0.1	/	> 0.1	14,66	↗+98	> 0.1	↗+35	> 0.1	9,81	↘-24	> 0.1	1
	Methylmalonic acid†	2,10	M-H-	117,01933	117,01917	1,4	29,45	/	> 0.1	/	> 0.1	/	> 0.1	6,07	/	> 0.1	/	< 0.05	25,94	/	> 0.1	1
	Pseudouridine	1,31	M-H-	243,06226	243,06153	3,0	ND	ND	ND	ND	ND	ND	ND	16,76	/	> 0.1	↗+40	< 0.05	19,25	↘-37	< 0.05	1
	Thymidine	2,39	M+FA-H-	287,08847	287,08832	0,5	14,77	↗+50	< 0.05	↘-42	< 0.05	/	> 0.1	7,66	↘-42	< 0.05	↘-48	< 0.05	3,55	/	> 0.1	1
	Thymine	1,92	M-H-	125,03565	125,03553	1,0	24,21	/	> 0.1	/	> 0.1	/	> 0.1	15,04	/	> 0.1	/	> 0.1	9,69	/	> 0.1	1
	Uracil†	1,52	M-H-	111,02000	111,01988	1,1	6,25	/	> 0.1	/	> 0.1	/	> 0.1	16,21	↘-40	< 0.05	/	> 0.1	5,90	/	> 0.1	1
	Ureidoisobutyric acid	1,65	M-H-	145,06187	145,06047	9,6	ND	ND	ND	ND	ND	ND	ND	40,16	↘-32	> 0.1	/	< 0.05	38,93	/	> 0.1	1
	Uridine	1,74	M+FA-H-	289,06774	289,06764	0,3	12,15	/	> 0.1	/	> 0.1	/	> 0.1	4,97	/	> 0.1	/	> 0.1	3,65	/	> 0.1	1
Pyruvate Metabolism	Acetic acid† (1)	1,22	M+FA-H-	105,01933	105,01920	1,2	40,84	/	> 0.1	/	> 0.1	/	> 0.1	5,52	/	< 0.05	/	> 0.1	2,71	/	> 0.1	1
	DL-Lactic Acid†	6,38	M-H-	89,02442	89,02438	0,4	8,10	/	> 0.1	/	> 0.1	↗+60	< 0.05	10,87	/	> 0.1	↗+23	< 0.05	5,37	↘-27	> 0.1	1
	Fumaric acid†	1,65	M-H-	115,00368	115,00356	1,1	62,36	/	> 0.1	/	> 0.1	/	> 0.1	3,66	/	< 0.1	/	> 0.1	89,01	/	> 0.1	1
	L-malic acid†	1,33	M-H-	133,01425	133,01402	1,7	3,82	/	> 0.1	/	> 0.1	/	> 0.1	3,09	/	< 0.05	/	> 0.1	4,74	/	< 0.05	1

Metabolism Pathway	Metabolite	Rt (min)	Adduct	Calculated mass	Observed mass	Δm/z (ppm)	RSD QC_M	M1		M3		M7		RSD QC_F	F1		F3		RSD QC	F7		Annotation level
								Diff Amp	pvalue	Diff Amp	pvalue	Diff Amp	pvalue		Diff Amp	pvalue	Diff Amp	pvalue		Diff Amp	pvalue	
Steroid hormone biosynthesis	4-Methylpentanal	10,07	M+ACN+H+	142,12264	142,12243	1,5	64,73	/	> 0.1	↗+124	< 0.05	/	> 0.1	9,93	/	> 0.1	/	> 0.1	9,32	/	> 0.1	1
	Cortolone	11,95	M+FA-H-	411,23883	411,23826	1,4	24,20	/	> 0.1	/	> 0.1	/	> 0.1	10,37	/	< 0.1	/	> 0.1	24,76	/	< 0.1	1
	Mevalonic acid	1,90	M-H-	147,06628	147,06607	1,4	38,13	/	> 0.1	/	> 0.1	/	> 0.1	6,37	↗+25	> 0.1	/	> 0.1	8,10	/	> 0.1	1
	Tetrahydrocortisol	12,24	M+FA-H-	411,23883	411,23790	2,2	36,67	↗+49	< 0.05	/	> 0.1	/	> 0.1	21,29	/	> 0.1	/	> 0.1	12,15	/	> 0.1	1
Tryptophan Metabolism	3-hydroxyanthranilic acid	5,53	M+H+	154,04987	154,04939	3,1	49,32	/	> 0.1	/	> 0.1	/	> 0.1	ND	ND	ND	ND	ND	ND	ND	ND	1
	5-Hydroxykynurenine	8,49	M+H+	225,08698	225,08675	1,0	8,00	↘-25	< 0.05	/	> 0.1	/	< 0.05	8,75	/	< 0.1	/	< 0.1	5,93	/	> 0.1	2
	Anthranilate	5,77	M+H+	138,05495	138,05461	2,5	20,46	/	> 0.1	/	> 0.1	/	> 0.1	4,71	/	> 0.1	/	> 0.1	8,54	↘-20	> 0.1	1
	DL-kynurenine	8,73	M+H+	209,09207	209,09197	0,5	7,68	/	> 0.1	/	> 0.1	/	> 0.1	9,18	/	< 0.05	↘-24	< 0.05	6,53	/	> 0.1	1
	Formylanthranilic acid	11,46	M-H-	164,03532	164,03499	2,0	8,63	/	> 0.1	/	> 0.1	/	> 0.1	3,67	↗+23	< 0.05	/	> 0.1	4,12	/	> 0.1	1
	Indolelactic acid	1,40	M+H+	206,08117	206,08296	-8,7	42,84	/	> 0.1	/	> 0.1	/	> 0.1	ND	ND	ND	ND	ND	ND	ND	ND	1
	Kynurenic acid	10,27	M+H+	190,04987	190,04922	3,4	36,51	↗+882	< 0.05	↗+126	> 0.1	/	< 0.05	30,65	↘-50	> 0.1	/	> 0.1	ND	ND	ND	1
	L-Tryptophan†	11,58	M+H+	205,09715	205,09700	0,7	6,00	/	> 0.1	↘-29	< 0.05	/	> 0.1	7,93	/	< 0.05	↘-46	< 0.05	26,28	/	> 0.1	1
	N'-Formylkynurenine	7,23	M-H-	235,07243	235,07182	2,6	ND	ND	ND	ND	ND	ND	ND	9,02	↘-36	> 0.1	↘-52	< 0.05	42,11	/	> 0.1	1
	Oxoadipic acid†	1,63	M-H-	159,02990	159,02996	-0,4	51,01	/	< 0.05	/	> 0.1	ND	ND	ND	ND	ND	ND	ND	ND	ND	ND	1
	Serotonin	10,46	M+H+	177,10224	177,10175	2,8	27,30	↘-36	> 0.1	/	> 0.1	↗+334	< 0.05	13,68	↘-63	< 0.05	↘-26	> 0.1	7,78	↗+95	< 0.1	1
Tyrosine Metabolism	1,2-Dehydrosalsolinol	9,84	M+H+	178,08625	178,08608	1,0	12,16	↗+31	> 0.1	↗+55	> 0.1	/	> 0.1	18,86	↗+84	> 0.1	↘-31	< 0.1	16,79	↗+87	> 0.1	2
	3-Hydroxyphenylacetic acid†	9,69	M-H-	151,04007	151,03971	2,4	5,47	/	> 0.1	/	> 0.1	/	> 0.1	15,54	/	> 0.1	/	> 0.1	5,06	/	> 0.1	1
	3,4-Dihydroxybenzeneacetic acid	5,67	M-H-	167,03498	167,03480	1,1	ND	ND	ND	ND	ND	ND	ND	23,22	/	> 0.1	/	> 0.1	103,85	/	> 0.1	1
	3,4-dihydroxymandelaldehyde	4,49	M-H-	167,03498	167,03462	2,2	45,02	/	> 0.1	↗+71	< 0.1	/	> 0.1	32,64	/	> 0.1	/	< 0.1	32,50	/	> 0.1	1
	4-Hydroxyphenylacetaldehyde	11,61	M-H-	135,04515	135,04517	-0,1	30,26	/	< 0.1	/	> 0.1	/	> 0.1	21,58	/	> 0.1	/	> 0.1	12,66	/	> 0.1	1
	4-Hydroxyphenylacetylglutamic acid	8,24	M+H+	282,09721	282,09697	0,9	6,51	↘-36	> 0.1	↘-20	> 0.1	↗+57	< 0.1	25,61	↘-49	< 0.1	/	> 0.1	4,88	/	> 0.1	2
	4-methoxytyramine	10,70	M+H+	168,10190	168,10149	2,5	ND	ND	ND	ND	ND	ND	ND	56,11	/	> 0.1	/	> 0.1	10,06	↗+56	> 0.1	1
	6-Hydroxydopamine	3,40	M+H+	170,08117	170,08075	2,5	16,57	↘-42	> 0.1	↗+50	> 0.1	/	> 0.1	43,64	/	> 0.1	/	> 0.1	ND	ND	ND	1
	Dopamine 4-sulfate	2,09	M-H-	232,02852	232,02826	1,1	64,71	/	> 0.1	↗+95	> 0.1	/	< 0.05	23,96	/	> 0.1	↘-27	> 0.1	40,91	/	> 0.1	1
	Epinephrine	3,67	M+H+	184,09682	184,09673	0,5	21,22	↗+88	< 0.05	/	> 0.1	↗+43	> 0.1	35,09	/	> 0.1	/	> 0.1	ND	ND	ND	1
	Fumaric acid†	1,65	M-H-	115,00368	115,00356	1,1	62,36	/	> 0.1	/	> 0.1	/	> 0.1	3,66	/	< 0.1	/	> 0.1	89,01	/	> 0.1	1
	Gamma-glutamyltyrosine	6,98	M-H-	309,10921	309,10871	1,6	7,04	/	> 0.1	/	> 0.1	/	> 0.1	6,64	↘-31	< 0.05	↘-20	< 0.05	4,29	↘-23	< 0.05	1
	Homovanillic acid	9,86	M-H-	181,05063	181,04992	3,9	ND	ND	ND	ND	ND	ND	ND	19,06	↗+22	> 0.1	/	> 0.1	69,36	/	> 0.1	1
	Hydroxyphenylacetylglycine	5,02	M-H-	208,06153	208,06126	1,3	36,39	↘-50	< 0.1	↗+73	< 0.1	/	> 0.1	ND	ND	ND	ND	ND	ND	ND	ND	1
	L-Tyrosine†	3,54	M+H+	182,08117	182,08109	0,4	3,48	/	> 0.1	↘-21	< 0.1	/	> 0.1	7,07	/	< 0.05	↘-37	< 0.05	7,35	/	> 0.1	1
	N-Acetyl-L-tyrosine	7,29	M-H-	222,07718	222,07692	1,2	5,10	/	> 0.1	/	> 0.1	/	> 0.1	18,46	↘-33	< 0.05	↘-27	< 0.05	4,00	/	> 0.1	1
	Norepinephrine	2,73	M-H-	168,06662	168,06636	1,6	24,58	/	> 0.1	/	> 0.1	/	> 0.1	17,27	/	< 0.05	/	> 0.1	8,69	/	> 0.1	1
	Normetanephrine	4,84	M-H2O+H+	166,08571	166,08594	-1,4	68,15	/	> 0.1	/	> 0.1	/	> 0.1	11,66	/	> 0.1	/	> 0.1	27,92	/	> 0.1	1
	O-tyrosine	7,40	M+H+	182,08117	182,08061	3,1	19,76	↗+37	< 0.1	↘-24	> 0.1	/	> 0.1	27,38	/	> 0.1	/	> 0.1	4,31	/	> 0.1	1
	P-octopamine	3,54	M-H2O+H+	136,07514	136,07558	-3,3	5,28	/	> 0.1	↘-21	< 0.1	/	> 0.1	5,31	/	< 0.05	↘-37	< 0.05	6,78	↘-33	< 0.05	1
	Phenol	7,81	M-H2O+H+	77,03803	77,03796	0,9	34,25	/	< 0.1	↘-46	< 0.05	/	> 0.1	17,62	/	< 0.1	↘-49	< 0.05	1,47	↘-31	> 0.1	2
	Tyramine	8,11	M+H+	138,09134	138,09120	1,0	15,36	/	> 0.1	↘-21	< 0.1	↗+30	> 0.1	21,72	/	> 0.1	/	> 0.1	25,93	/	> 0.1	1
Valine, leucine and isoleucine Metabolism and derivatives	3-Methyl-2-oxovaleric acid	7,05	M-H-	129,05572	129,05557	1,1	42,03	/	> 0.1	/	> 0.1	/	> 0.1	26,41	ND	ND	/	> 0.1	15,67	/	> 0.1	1
	DL-isoleucine or L-Alloisoleucine or L-Isoleucine or L-Leucine	3,92	M+H+	132,10190	132,10175	1,2	68,62	/	> 0.1	/	> 0.1	/	< 0.05	18,82	↘-26	< 0.05	/	> 0.1	3,19	↘-22	> 0.1	1
	Erythronilic acid	2,71	M-H20-H-	99,04460	99,04504	-4,4	9,16	↗+60	> 0.1	↘-28	> 0.1	/	> 0.1	5,61	↗+48	< 0.1	/	> 0.1	4,90	/	> 0.1	2
	Hydroxyisocaproic acid	8,74	M-H-	131,07137	131,07114	1,8	2,29	/	> 0.1	↗+125	< 0.05	/	> 0.1	2,36	↘-40	< 0.05	↘-22	> 0.1	78,98	/	> 0.1	1
	L-Valine	2,69	M+H+	118,08625	118,08606	1,6	6,42	/	> 0.1	/	> 0.1	/	> 0.1	67,03	/	< 0.05	/	< 0.05	50,61	↗+89	> 0.1	1
	Methylmalonic acid†	2,10	M-H-	117,01933	117,01917	1,4	29,45	/	> 0.1	/	> 0.1	/	> 0.1	6,07	/	> 0.1	/	< 0.05	25,94	/	> 0.1	1
	N-acetylisoleucine	9,44	M-H-	172,09792	172,09771	1,2	4,41	/	> 0.1	/	> 0.1	/	> 0.1	2,30	↘-34	< 0.05	↘-30	< 0.05	4,96	/	> 0.1	1
	N-Acetylvaline	4,80	M-H-	158,08227	158,08198	1,8	11,99	↘-29	< 0.05	/	> 0.1	/	> 0.1	19,66	↘-35	< 0.05	/	> 0.1	11,07	/	> 0.1	1
	Pyridoxal†	3,29	M+H+	168,06552	168,06556	-0,2	ND	ND	ND	ND	ND	ND	ND	40,24	/	> 0.1	/	> 0.1	30,99	/	> 0.1	1

Metabolism Pathway	Metabolite	Rt (min)	Adduct	Calculated mass	Observed mass	Δm/z (ppm)	RSD QC_M	M1		M3		M7		RSD QC_F	F1		F3		RSD QC	F7		Annotation level
								Diff Amp	pvalue	Diff Amp	pvalue	Diff Amp	pvalue		Diff Amp	pvalue	Diff Amp	pvalue		Diff Amp	pvalue	
Vitamin B6 metabolism	4-Pyridoxic acid	1,44	M-H-	182,04588	182,04566	1,2	7,38	/	> 0.1	↘-46	< 0.1	/	> 0.1	2,04	/	> 0.1	/	> 0.1	3,63	/	> 0.1	1
	5-Pyridoxolactone	3,25	M-H-	164,03532	164,03500	1,9	8,12	/	> 0.1	↗+24	> 0.1	/	> 0.1	5,94	↗+26	< 0.1	/	> 0.1	1,47	/	> 0.1	2
	L-Glutamine†	1,26	M+H+	147,07642	147,07623	1,3	10,01	/	> 0.1	↘-23	> 0.1	/	< 0.05	5,26	/	> 0.1	↘-38	< 0.05	9,98	↘-33	< 0.05	1
	Pyridoxal†	3,29	M+H+	168,06552	168,06556	-0,2	ND	ND	ND	ND	ND	ND	ND	40,24	/	> 0.1	/	> 0.1	30,99	/	> 0.1	1
	Pyridoxine	10,81	M-H20-H-	150,05550	150,05581	-2,1	31,43	/	> 0.1	/	> 0.1	/	< 0.1	16,81	↗+66	< 0.05	/	> 0.1	10,92	↗+24	> 0.1	2
Other metabolites	2-Aminoisobutyric acid	1,55	M-H-	102,05605	102,05577	2,7	4,86	/	> 0.1	/	< 0.1	/	> 0.1	65,88	/	> 0.1	/	< 0.05	ND	ND	ND	1
	2-Ketohexanoic acid	3,56	M+H+	131,07027	131,07023	0,3	39,56	/	> 0.1	/	> 0.1	/	> 0.1	12,72	↗+49	< 0.1	/	> 0.1	9,85	/	> 0.1	2
	2-Methylglutaric acid	4,46	M-H-	145,05063	145,05034	2,0	5,21	↗+66	> 0.1	↘-31	> 0.1	/	> 0.1	16,01	/	< 0.1	/	> 0.1	19,42	/	> 0.1	1
	4-Aminobutyraldehyde	1,37	M+FA-H-	132,06661	132,06641	1,6	19,38	↘-21	> 0.1	/	> 0.1	/	> 0.1	28,55	/	> 0.1	/	< 0.05	32,51	/	> 0.1	2
	8-Methylnonenoate	12,93	M+FA-H-	215,12888	215,12845	2,0	64,21	/	> 0.1	/	> 0.1	/	> 0.1	45,42	/	> 0.1	/	> 0.1	8,18	/	> 0.1	1
	Acetolactate	1,21	M-H-	131,03498	131,03466	2,5	57,49	/	< 0.05	/	< 0.1	/	> 0.1	34,69	/	> 0.1	/	< 0.05	28,73	/	> 0.1	1
	Ascorbic acid	1,31	M-H-	175,02481	175,02594	-6,4	ND	ND	ND	ND	ND	ND	ND	12,10	/	> 0.1	/	> 0.1	4,80	/	> 0.1	1
	Ethyladipic acid	10,35	M-H-	173,08193	173,08160	1,9	5,94	↗+81	< 0.1	↘-28	> 0.1	/	> 0.1	3,18	↗+40	< 0.1	↗+26	> 0.1	2,68	/	> 0.1	2
	Gamma glutamyl ornithine	3,19	M-H2O+H+	244,12863	244,12905	-1,7	ND	ND	ND	ND	ND	ND	ND	34,59	/	> 0.1	/	< 0.05	31,46	↘-53	< 0.05	2
	Indoleacrylic acid	11,58	M+H+	188,07060	188,07043	1,0	21,35	/	> 0.1	/	< 0.05	/	> 0.1	6,94	/	< 0.05	↘-47	< 0.05	8,12	/	> 0.1	2
	L-Acetylcarnitine	5,78	M+H+	204,12303	204,12283	1,0	4,28	/	> 0.1	/	> 0.1	↗+63	< 0.1	7,76	↘-33	> 0.1	/	> 0.1	37,57	/	> 0.1	1
	L-Carnitine	2,68	M+H+	162,11247	162,11228	1,1	3,90	/	> 0.1	/	> 0.1	/	> 0.1	22,58	/	> 0.1	/	< 0.1	10,78	/	> 0.1	1
	L-homocysteic acid	1,02	M-H-	182,01287	182,01254	1,8	2,44	/	> 0.1	/	> 0.1	/	> 0.1	3,03	/	> 0.1	/	> 0.1	0,98	/	> 0.1	1
	N-acetyl-L-alanine◊	1,81	M-H-	130,05097	130,05079	1,4	13,27	/	> 0.1	/	> 0.1	/	< 0.05	1,91	/	< 0.05	/	< 0.05	3,37	/	> 0.1	1
	N-alpha-Acetyl-L-lysine	2,60	M+H+	189,12337	189,12311	1,4	33,09	/	> 0.1	/	> 0.1	/	> 0.1	43,17	/	> 0.1	/	< 0.05	15,58	/	> 0.1	1
	N-alpha-acetylcitrulline	1,47	M-H-	216,09898	216,09843	2,5	ND	ND	ND	ND	ND	ND	ND	ND	ND	ND	ND	ND	15,13	↘-22	< 0.05	1
	O-Propanoyl-D-carnitine	10,38	M+H+	218,13868	218,13854	0,7	9,90	↗+172	> 0.1	↗+478	> 0.1	↗+779	< 0.1	14,08	↗+25	> 0.1	/	> 0.1	15,46	↘-40	> 0.1	2
	Phosphate	0,98	M+FA-H-	142,97510	142,97481	2,0	8,69	/	> 0.1	/	> 0.1	/	> 0.1	3,28	/	> 0.1	/	> 0.1	1,96	/	> 0.1	2
	Phosphoglycolic acid	0,93	M-H-	154,97510	154,97536	-1,7	50,55	↗+78	> 0.1	/	> 0.1	/	> 0.1	ND	ND	ND	ND	ND	24,35	/	> 0.1	1
	Phtalic acid	9,31	M-H-	165,01933	165,01904	1,8	6,44	/	> 0.1	/	> 0.1	↗+84	< 0.05	20,39	/	> 0.1	/	> 0.1	3,54	/	> 0.1	1
	Pyrrolidin-2-one	1,85	M+Na+	108,04198	108,04183	1,4	7,98	↗+147	< 0.05	/	> 0.1	↘-22	> 0.1	9,66	/	> 0.1	/	> 0.1	14,47	/	> 0.1	1
	Salsoline-1-carboxylate	9,94	M+H+	238,10738	238,10709	1,2	14,57	↘-28	< 0.1	/	> 0.1	/	> 0.1	13,13	↘-24	< 0.05	/	> 0.1	8,50	/	> 0.1	2
	Salsolinol 1-carboxylate	3,15	M+FA-H-	268,08266	268,07997	10,0	36,24	/	< 0.05	/	> 0.1	/	< 0.05	ND	ND	ND	ND	ND	ND	ND	ND	1
	Tetradecandioic acid	14,19	M-H-	257,17583	257,17552	1,2	3,49	/	> 0.1	/	> 0.1	/	> 0.1	3,61	/	< 0.1	/	< 0.1	9,72	/	> 0.1	1
	Vanillactic acid	8,64	M-H-	211,06120	211,06092	1,3	14,52	↗+39	> 0.1	↘-28	> 0.1	/	> 0.1	8,97	/	> 0.1	/	> 0.1	16,73	/	> 0.1	1
	Vanillic acid 4-sulfate	10,65	M+FA-H-	293,11430	293,11410	0,7	7,21	/	> 0.1	/	> 0.1	↘-25	< 0.1	6,54	↘-38	< 0.05	↘-26	< 0.05	4,96	↘-37	< 0.05	2
	Vanillylamine	11,75	M+H+	154,08625	154,08603	1,4	20,71	↗+43	> 0.1	↘-34	> 0.1	/	> 0.1	11,40	↗+24	> 0.1	/	> 0.1	8,52	/	> 0.1	2

Figure S1*: Quantitative enrichment analysis for male mussels exposed to 10 µg/L of venlafaxine after 1 day (A), 3 days (B) and 7 days (C). Red lines and rectangles represent pathways with a p-value < 0.1, blue lines and rectangles represent pathways with p-value < 0.05.*

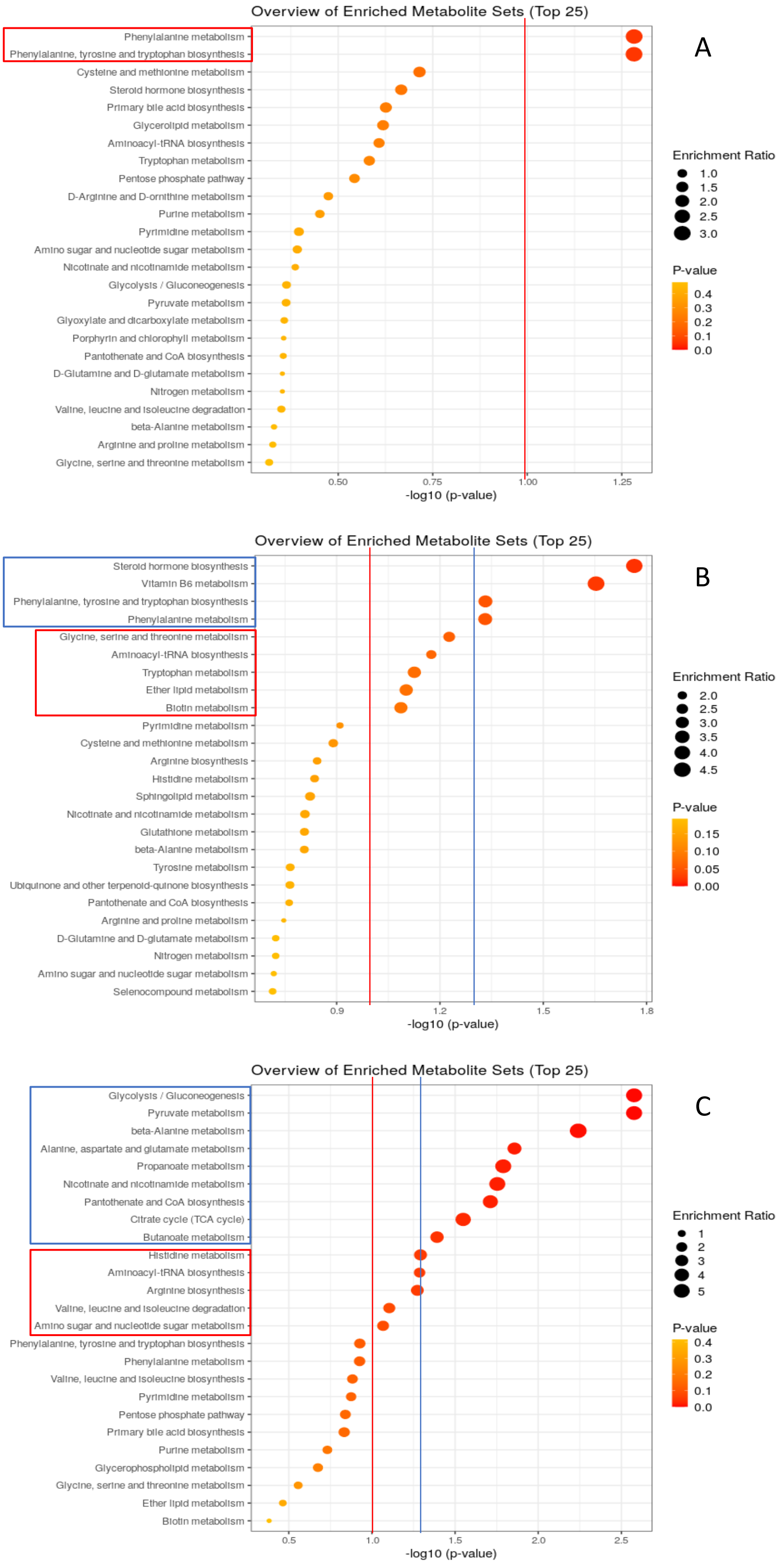

***Figure S2**: Quantitative enrichment analysis for female mussels exposed to 10 µg/L of venlafaxine after 1 day (A), 3 days (B) and 7 days (C). Red lines and rectangles represent pathways with a p-value < 0.1, blue lines and rectangles represent pathways with p-value < 0.05.*

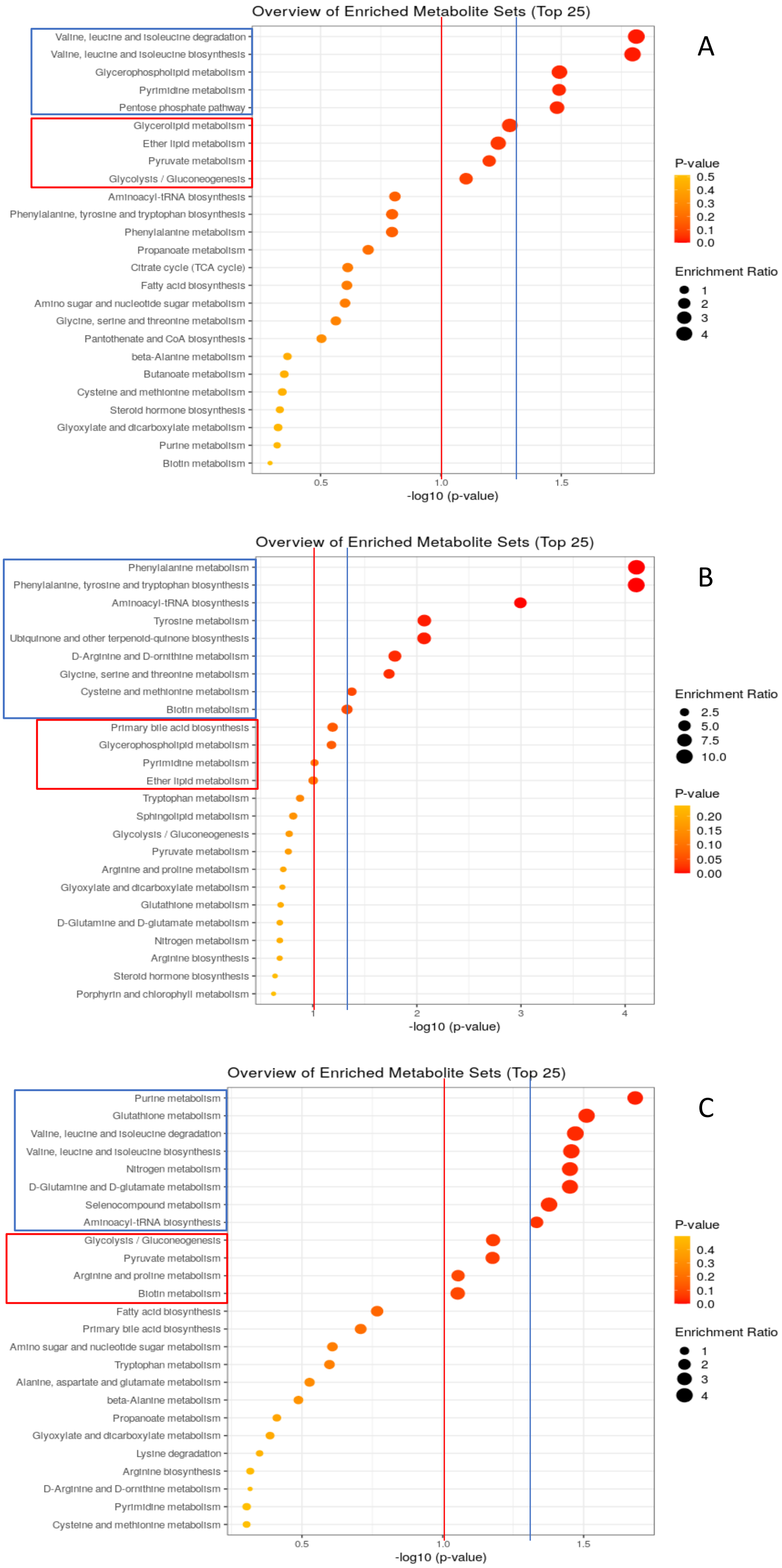

Supplement: Supplementary file 1 [file metabolites-12-00197-s001.zip › metabolites-1539970-supplementary.pdf]
